# Antagonism of STAT1 by Nipah virus P gene products modulates disease course but not lethal outcome in the ferret model

**DOI:** 10.1038/s41598-019-53037-0

**Published:** 2019-11-13

**Authors:** Benjamin A. Satterfield, Viktoriya Borisevich, Stephanie L. Foster, Sergio E. Rodriguez, Robert W. Cross, Karla A. Fenton, Krystle N. Agans, Christopher F. Basler, Thomas W. Geisbert, Chad E. Mire

**Affiliations:** 1 Galveston National Laboratory, University of Texas Medical Branch, Galveston, TX USA; 2 Department of Microbiology and Immunology, University of Texas Medical Branch, Galveston, TX USA; 3 Mayo Clinic, Department of Medicine, Rochester, MN USA; 4 Center for Microbial Pathogenesis, Institute for Biomedical Sciences, Georgia State University, Atlanta, GA USA

**Keywords:** Microbiology, Viral immune evasion

## Abstract

Nipah virus (NiV) is a pathogenic paramyxovirus and zoononis with very high human fatality rates. Previous protein over-expression studies have shown that various mutations to the common N-terminal STAT1-binding motif of the NiV P, V, and W proteins affected the STAT1-binding ability of these proteins thus interfering with he JAK/STAT pathway and reducing their ability to inhibit type-I IFN signaling, but due to differing techniques it was unclear which amino acids were most important in this interaction or what impact this had on pathogenesis *in vivo*. We compared all previously described mutations in parallel and found the amino acid mutation Y116E demonstrated the greatest reduction in binding to STAT1 and the greatest reduction in interferon antagonism. A similar reduction in binding and activity was seen for a deletion of twenty amino acids constituting the described STAT1-binding domain. To investigate the contribution of this STAT1-binding motif in NiV-mediated disease, we produced rNiVs with complete deletion of the STAT1-binding motif or the Y116E mutation for ferret challenge studies (rNiV_M_-STAT1^blind^). Despite the reduced IFN inhibitory function, ferrets challenged with these rNiV_M_-STAT1^blind^ mutants had a lethal, albeit altered, NiV-mediated disease course. These data, together with our previously published data, suggest that the major role of NiV P, V, and W in NiV-mediated disease in the ferret model are likely to be in the inhibition of viral recognition/innate immune signaling induction with a minor role for inhibition of IFN signaling.

## Introduction

Nipah virus (NiV; family *Paramyxoviridae*) emerged in Southeast Asia two decades ago as a novel, lethal human pathogen. NiV infection results in acute respiratory disease, meningoencephalitis, and multiorgan vasculitis^1,2^. Overall mortality is high, with some outbreaks reaching nearly 100%^3,4^. The minority of patients that do survive have high rates of persistent neurological sequelae^5^. A single outbreak occurred in Malaysia and Singapore^6^, an outbreak was reported in the Philippines^7^, while frequent outbreaks have been recorded since the discovery of NiV in Bangladesh and India^8–13^.

The ferret model of NiV infection recapitulates NiV infection in humans^2,14,15^. Therefore, the ferret has been extensively used to study NiV pathogenic mechanisms, as well as to assess the protective effectiveess of experimental vaccines and therapeutics^14–23^. With the development of a reverse genetics system by several groups^20,21,23–28^ multiple aspects of NiV pathogenesis have become more clear including the contribution of the C and W proteins in NiV-mediated respiratory disease^21^ and the primary contribution of the V protein leading to lethality^20^. NiV inhibits interferon (IFN) signaling through Signal Transducer and Activator of Transcription (STAT) antagonism as determined in plasmid overexpression studies^24,29–31^ and *in vitro*^24,32^, but the relevance of STAT1 inhibition by NiV proteins *in vivo* has not yet been described.

IFN-α and -β are involved in innate immune control of viral infections by signaling cells through the Janus Kinase (JAK)/STAT pathway which results in increased expression of numerous IFN-stimulated genes (ISGs). Many viruses deploy mechanisms of inhibiting these signaling cascades and the production of IFN^33^. In paramyxoviruses, this is typically accomplished through the P gene products^34,35^. Four genes are encoded by the NiV P gene (P, V, W, and C)^36^; of these, P, V, and W share an N-terminal amino acid sequence which binds STAT1 inhibiting its activation through phosphorylation^31^. NiV P, V, and W all sequester STAT1 after binding to it, however, P and V sequester STAT1 in the cytoplasm while W sequesters STAT1 within the nucleus, although perhaps not in all cell types^37^. STAT1 inhibition is not the only mechanism of IFN antagonism demonstrated by NiV; the V protein can inhibit STAT2^38^, RIG-I^39^, and MDA5^40^ while the W protein blocks signaling through both TANK-binding kinase 1 (TBK1) and Inhibitor of κB kinase ε (IKKε)^41^. The function of NiV C remains elusive. It does interfere to some degree with viral RNA synthesis^32,36,42^ leading to a weakening of type I IFN induction. NiV C protein has also been reported to bind IKKα, thus antagonizing TLR7/9-dependent IFN-α induction^43^.

Several previous studies localized the STAT1-binding domain to amino acids 114–140 of the P protein (also shared with V and W); remarkably, deletion of this region does not alter the effect the genome replication function of P^24,31^. Three previous studies have identified seven amino acids within this domain that decrease STAT1-binding and/or inhibition of IFN signaling when mutations were introduced^24,29,30^. These amino acid substitutions consist of Y116E, G121E, G127E, and G135E^24^; G125E^24,29^; and S130A and S131A^30^.

Using reverse genetics, two *in vitro* studies have examined single amino acid mutations, namely G121E^24^ and G125E^32^, in this STAT1-binding domain. The STAT1-binding domain overlaps with the open reading frame (ORF) of the C protein and mutations introduced to this region also necessitate amino acid substitutions in C. One strategy to prevent confounding results is to produce rNiV mutants in the context of a C protein knock-out (C^ko^) backbone, which was the strategy employed in one study examining the G121E mutation with a C^ko^ mutant rNiV used in place of a wild-type rNiV^24^. This study showed that the G121E mutation prevented STAT1 phosphorylation and sequestration in infected cells demonstrating that this is not an artifact of a plasmid over-expression system. A second study engineered G125E in a wild-type (not C^ko^) backbone^32^. Compared with rNiV_M_-wild-type (wt) infection, cells infected with this rNiV_M_-P_G121E_ increased early ISG production, however not increased production of IFN-β, Interferon Gamma-Induced Protein 10 (IP-10), or Regulated on Activation Normal T Cell Expressed and Secreted (RANTES), thus suggesting that production of IFN and, subsequently, the role of the STAT1-binding domain might have minimal impact in NiV infection.

The present study has a side-by-side comparison of all seven described mutations in the STAT1-binding region. The most potent single amino acid mutation and a deletion of the entire STAT1-binding region were then introduced in rNiVs and the role of this STAT1 antagonism was then examined in the ferret model. This study demonstrates that the level of NiV STAT1 antagonism plays a minor role in modulating disease course but is not necessary for a lethal outcome.

## Materials and Methods

### Cell lines

As previously described^20^, BSR-T7/5 cells, a BHK-21 cell line stably expressing T7 RNA polymerase^44^, were maintained in Dulbecco’s modified Eagle medium (DMEM; Gibco, Carlsbad, CA) supplemented with 10% fetal bovine serum (FBS; Gibco), 100 U/ml penicillin, 100 g/ml streptomycin, and 0.5 mg/ml Geneticin (Gibco). Vero 76 cells (ATCC CRL-1587) were maintained in Eagle’s Minimum Essential Medium (EMEM) supplemented with 10% FBS and 100 U/ml penicillin (Gibco), 100 g/ml streptomycin (Gibco). HEK 293 T/17 cells (ATCC CRL-11268) were maintained in DMEM supplemented with 10% FBS, 100 U/ml penicillin and 100 g/ml streptomycin.

### Expression plasmids

Constitutively expressed pCAGGS-HA NiV_M_ P, pCAGGS-HA NiV_M_ V, and pCAGGS-HA NiV_M_ W plasmids had been previously constructed^24,31,41^; briefly, the P, V, or W gene was hemagglutinin (HA)-tagged at the amino terminus and subcloned into the pCAGGS expression plasmid. The following mutations were introduced into each of the pCAGGS-HA NiV_M_ P, V, and W expression plasmids: Y116E (T2751A and C2753G), G121E (G2767A), G125E (G2779A), G127E (G2785A), S130A (T2793G and A2795C), S131A (A2796G and G2897C), and G135E (G2809A and G2810A) either individually or in combination; Δ121–130 (deletion of nucleotides 2766 to 2795), and Δ116–135 (deletion of nucleotides 2751 to 2810); all site-directed mutagenesis was performed by Mutagenex Inc. (Piscataway, NJ). The constitutively expressed pCAGGS-STAT1-GFP plasmid was described previously^24,45^, the pRL-CMV (Promega) plasmid constitutively expresses Renilla luciferase, and the constitutively expressed pISG54-firefly luciferase plasmid was described previously^24^.

### Plasmid construction and generation of recombinant NiVs

As previously describe^20^, the NiV genomic sequence used to construct the rNiVs in this study was UMMC1 (GenBank accession no. AY029767), an isolate cultured from the cerebrospinal fluid of an encephalitic human patient in the initial outbreak in Malaysia. This NiV_M_ genome was assembled into three segments, A (nt 1-6,780), B (nt 6,780-10,404), and C (nt 10,404-18,246) as described previously^20,24^. These fragments could then be mutated followed by assembly into full-length cDNA clones in pSL1180 cloning vectors containing T7 promoter and terminator sequences and a hepatitis delta virus ribozyme sequence^20^. Two NiV_M_ full-length cDNA clones (pFL-NiV_M_-P_Y116E_ and pFL-NiV_M_-P_Δ116-135_) were constructed. Site-directed mutagenesis was performed by Mutagenex Inc. to introduce the P_Y116E_ or P_Δ116-135_ mutations described above in the A segment; these mutations disrupt STAT1 binding of the NiV P, V, and W proteins.

The pTM1-HA NiV_M_ P helper plasmid was constructed as described previously^20,24^; briefly, the P gene was HA-tagged at the amino terminus and subcloned into the pTM1 expression plasmid. The pTM1.W-NiV_M_ N and pTM1.W-NiV_M_ L helper plasmids were constructed previously^20^ by amplifying the sequences for the N and L genes from the A and C segments, respectively, with PCR and were cloned into a pTM1.W expression vector.

rNiV_M_ mutants were generated as described previously^20^. Briefly, BSR-T7/5 cells were seeded in six-well plates and were co-transfected with 3.5 µg of NiV_M_ full-length cDNA clone, 0.2 µg of pTM1-HA NiV_M_ P, 0.75 µg of pTM1.W-NiV_M_ N, 0.4 µg of pTM1.W-NiV_M_ L per well in Optimem (Gibco) with Lipofectamine 2000 (Invitrogen, Carlsbad, CA) according to manufacturer’s protocol. At 72 hours post-transfection the medium and cells were collected and passaged onto Vero cells. Cytopathic effect (CPE) was typically observed beginning between days 4 and 8 post infection (p.i.) The medium was then collected and passed on Vero cells for plaque purification of the virus. A small quantity (passage 1 [P1]) of the plaque purified virus was then grown in Vero cells followed by a larger quantity (P2) in Vero cells infected with a multiplicity of infection (MOI) of 0.01. At 48 hours p.i. the virus-containing medium was harvested, clarified by low-speed centrifugation, aliquoted, and stored at −80 °C. The presence of introduced mutations was confirmed by deep sequencing viral RNA from the P2 stock. Virus titers were determined by standard plaque assay using 5% neutral red as described previously^20^. Experiments utilizing full-length clones or infectious rNiV_M_ were conducted with approved protocols in biosafety level 4 (BSL-4) containment at the Galveston National Laboratory (GNL) in Galveston, TX.

### IFN-induced luciferase expression assays

Wells were plated with 1.2 × 10^6^ 293 T cells per well and transfected with 500 ng of pCAGGS-HA NiV_M_ P, V, or W expression plasmids with or without the described mutations using Lipofectamine LTX (Invitrogen) per manufacturer’s protocol. After 24 hours samples were collected in 2X Laemmli sample buffer (Bio-Rad) and heated to 95 °C for 10 minutes. As described previously^20^, samples were then run on a denaturing 4–12% SDS-PAGE gel (Bio-Rad) and proteins were transferred from the gel onto polyvinylidene fluoride (PVDF) membranes and blocked in TTBS (100 mM Tris-HCl pH 7.5, 0.9% NaCl, 0.1% Tween 20) with 5% skim milk. PVDF membranes were incubated with polyclonal rabbit antisera against the unique C-terminal domains of the NiV P, W and V proteins. The antisera were produced by GenScript (Piscataway, NJ) as described previously^20^ diluted in TTBS with 5% milk (P: 1:5,000; V: 1:500; W: 1:500) for 1 hour at room temperature, and washed 4 times in TTBS. The membranes were then incubated with anti-rabbit IgG conjugated to horseradish peroxidase (HRP; Thermo Scientific, Waltham, MA; dilution 1:5,000) for 1 hour at room temperature, washed 4 times in TTBS, incubated with ECL reagent (Promega) for 5 minutes, and imaged with a VersaDoc (Bio-Rad). The relative level of each protein was quantified using the NIH’s open access ImageJ software and ratios determined compared to the wild-type P, V, or W protein expression. These ratios were used to slightly modify the amount of each mutant P, V, or W expression plasmid in the following experiments.

For transfection experiments 4 × 10^4^ 293 T cells were plated in 96-well plates. After 24 hours, each well was transfected with 25 ng pRL-CMV, 100 ng of pISG54-firefly luciferase, and about 300 ng (amounts were previously optimized and then modified by above ratios) of the pCAGGS-HA NiV_M_ P, V, or W expression plasmids with or without the described mutations. 300 ng of vector only and expression plasmids producing NiV_M_ N or L were used as controls. Transfections were performed with Lipofectamine LTX according to the manufacturer’s protocol. After 24 hours, 1000 U/ml of universal IFN-α was added to the medium. After 18 hours, a Dual-Glo luciferase assay system (Promega) was used to measure luciferase activity according to the manufacturer’s protocol in a Tecan Infinite M200 Pro (Tecan Group Ltd.). Transfection experiments were carried out in triplicate wells, and with each experiment being performed two or more times. Normalization with a firefly luciferase activity to Renilla luciferase activity ratio was performed to account for variations in transfection efficiency.

### Co-immunoprecipitation and Western blot analysis

HEK293T cells were plated in six-well plates to be transfected at 80 to 90% confluency the next day. Cells were transfected with 2.0 µg each of pCAGGS-STAT1-GFP and pCAGGS-HA NiV_M_ P, V, or W with or without the described mutations, using Lipofectamine 2000. Briefly, plasmids and transfection reagent (12 µl/well) were added to Opti-MEM (Gibco) and incubated at room temperature before addition to cells. After three hours, the transfection mixture was removed and complete media containing 10% FBS was added to cells.

At 24 hours post-transfection, cells were lysed with 300 µl of Mammalian Protein Extraction Reagent (M-PER; Thermo Scientific) according to manufacturer protocol. Protein was quantified using a bicinchoninic acid (BCA) Protein Assay Kit (Thermo Scientific). Lysates were standardized to the sample with the lowest concentration, and, for immunoprecipitation, equal amounts of protein (µg) were incubated with anti-HA agarose beads (HA-Tag IP/Co-IP Kit; Thermo Scientific) at 4 °C overnight and then prepared for SDS-PAGE according to the manufacturer’s protocol. Similarly, equivalent amounts of whole cell lysates (µg) were treated with 5X Laemmli sample buffer containing β-mercaptoethanol and heated to 95 °C for 5 minutes.

Equivalent volumes of immunoprecipitation samples, and 5 µg of whole cell lysate samples per well, were run on denaturing 7.5% polyacrylamide gels and then transferred to PVDF membranes (Bio-Rad). Membranes were blocked in Tween-Tris-buffered saline (TTBS) with 5% skim milk or bovine serum albumin (BSA; Fisher Scientific, Fair Lawn, NJ) according to antibody manufacturer recommendations. Membranes were incubated with polyclonal chicken antiserum against HA (dilution 1:5,000; Abcam, Cambridge, UK) or monoclonal rabbit antisera against STAT1-alpha (dilution 1:1,000; Abcam) diluted in TTBS with 5% milk or BSA overnight at 4 °C, followed by 4 washes in TTBS. Membranes were then incubated with anti-rabbit IgG conjugated to HRP (dilution, 1:5,000; Thermo Scientific) for 2 hours at room temperature, followed by 4 washes in TTBS. All membranes were incubated with SuperSignal West Pico substrate for 2 minutes and imaged with a VersaDoc (Bio-Rad).

Binding efficiency was evaluated by measuring the amount of STAT1 that co-immunoprecipitated with NiV P, V, or W wt or mutant proteins using densitometry assessed by ImageJ software (NIH). These values were then normalized to the total STAT1 levels for the same well also assessed by densitometry. HA blots showing NiV proteins and total protein gels were run as controls. Co-immunoprecipitation and subsequent Western blots with densitometry measurements were performed in triplicate with the means and standard error of the means presented.

### Immunofluorescence assays

Immunofluorescence assay analysis was carried out by Lipofectamine 2000 transfections of 80–90% confluent 293 T cell monolayers on glass coverslips with co-optimized plasmid concentrations of 1.5 μg of pCAGGS-STAT1-GFP and 1.0 μg of one of the pCAGGS-HA NiV_M_ P, V, or W expression plasmids with or without the described mutations. Following 18 hours of transfection, cells were exposed to 1,000 U/ml of universal IFN-α (PBL Assay Science, Piscataway, NJ) for 1 hour. Cells were then fixed with 4% (w/v) paraformaldehyde (Electron Microscopy Sciences, Hatfield, PA) and permeabilized with 1% Triton X-100. Cells were washed, blocked with 3% bovine serum albumin, and incubated overnight with a dilution of 1:1,000 Alexa Fluor 594 conjugated mouse anti-HA (HA.11; Invitrogen). Coverslips with stained cells were mounted onto glass slides and imaged on a Nikon Eclipse Ti-S fluorescence microscope (Nikon Instruments Inc., Melville, NY) using FITC filter (465–495 nm excitation), Texas Red filter (590–650 nm excitation), and X-Cite LEipD light drive (Lumen Dynamics, Ontario, Canada).

### Virus growth kinetics

Virus growth kinetics were measured as previously described^20^, briefly 1.2 × 10^6^ cells per well of Vero cells were seeded in six-well plates and incubated at 37 °C for 12 hours with either regular medium, or medium containing 1000 U/ml of universal IFN-α. The cells were then infected at an MOI of 0.01 with rNiV_M_-wt, rNiV_M_-P_Y116E_, rNiV_M_-P_Δ116–135_, or rNiV_M_-C^ko^ for 1 hour followed by the removal of the inoculum, 4 washes with PBS, and the addition of fresh medium. Supernatants were collected at 1, 6, 12, 24, 36, 48, and 72 hours p.i., clarified by centrifugation, aliquoted, and stored at −80 °C. All infections were performed in duplicate. Samples were then titered on Vero cells using standard plaque assays. Limit of detection was 25 PFU/ml.

### Statistics

Certain limitations are inherent when conducting animal studies in a BSL-4 environment including the need to restrict the number of animals used and the amount of biological samples obtained and stored. These limits in some cases preclude multiple independent repetitions and thus limits statistical analysis. Therefore, *in vivo* data are presented as the mean calculated from replicate animals or animal samples, not replicate assays.

Prism 5 (Graphpad Software, Inc., San Diego, CA) software was used to generate all graphs and to calculate statistical significance throughout this study using the following tests: one-way analysis of variance (ANOVA) followed by Tukey’s post-hoc test for co-immunoprecipitation Western blots; ANOVA with Dunnett’s multiple comparison test for viral growth kinetics and chemokine/cytokine analysis; Log-rank (Mantel-Cox) Test for Kaplan-Meier survival curves.

### STAT1 sequence analysis

Amino acid sequences of the STAT1 gene from humans (assession number NP_009330.1) and ferrets (assession number XP_012917318.1) were compared using NCBI’s BLASTp software (https://blast.ncbi.nlm.nih.gov/).

### Animals

BSL-4 biocontainment at the GNL located at the University of Texas Medical Branch (UTMB) was used for all animal studies and they were approved by the UTMB Institutional Animal Care and Use Committee (IACUC) which ensured that all research was compliant with federal statutes and regulations, including the Animal Welfare Act and complied with the principles outlined in the *Guide for the Care and Use of Laboratory Animals*, National Research Council, eighth edition, 2011^46^. The GNL is fully accredited by the Association for Assessment and Accreditation of Laboratory Animal Care International.

As previously described^20^, fifteen female, 6–8-month-old ferrets (*Mustela putorius furo*) weighing 0.75–1.0 kg were housed in groups of 2 or 3 animals per virus cohort. Before infection, subjects were anesthetized by 5% isofluorane and had transponder chips (BioMedic Data Systems, Seaford, DE) implanted subcutaneously for animal identification and temperature monitoring. For challenge and procedures, animals were anesthetized with a ketamine acepromazine xylazine (KAX) cocktail and inoculated intranasally (i.n.) with 5 × 10^3^ PFU of rNiV_M_-wt, rNiV_M_-P_Y116E_, rNiV_M_-P_Δ116–135_, in 0.5 ml of 2% FBS Hank’s Balanced Salt Solution (HBSS; Gibco). As previously described^20^, animals were anesthetized for clinical examination, respiration quality, and blood collection on days 0, 3, 6, and 10 p.i. and terminal endpoint. After challenge animals were assessed daily for weight, temperature, and scored on a scale of 0–12 for clinical observations based on coat appearance, social behavior, and provoked behavior; animals scoring 9 or greater were euthanized per UTMB IACUC protocol.

### rNiV_M_ serum neutralization assays

PRNT_50_s were determined using a conventional serum neutralization assay as previously described^20^. Briefly, sera were serially diluted twofold, and incubated with ~100 PFU of rNiV_M_-wt for 1 hour at 37 °C. Virus and antibodies were then added to individual wells of 6-well plates of confluent Vero cell monolayers in duplicate. Plates were stained with neutral red 2 days after infection and plaques were counted 24 hours after staining. The 50% neutralization titer (PRNT_50_) was determined as the serum dilution at which there was a 50% reduction in plaque counts versus control wells.

### Collection and processing of specimens from rNiV_M_-infected ferrets

As previously described^20^, blood was collected and placed in MiniCollect ethylenediaminetetraacetic acid (EDTA) tubes or serum tubes (Greiner Bio One, Monroe, NC). Immediately following sampling, 100 μl of blood was added to 600 μl of AVL viral lysis buffer with carrier RNA (Qiagen) for RNA extraction. For tissue sample processing, approximately 100 mg was stored in 1 ml RNAlater (Qiagen) for 96 hours to stabilize RNA. RNAlater was completely removed, and tissues were homogenized in 600 μl RLT buffer (Qiagen) in a 2-ml cryovial using a tissue lyser (Qiagen) and 1.4 mm ceramic beads (Precellys; Bertin Corp., Saint-Quentin-en-Yvelines Cedex, France). The tissues sampled included right lung upper lobe, right lung middle lobe, right lung lower lobe, left lung upper lobe, left lung middle lobe, left lung lower lobe, liver, spleen, kidney, adrenal gland, pancreas, and brain (frontal cortex). All blood samples were inactivated in AVL viral lysis buffer with carrier RNA, and tissue samples were homogenized and inactivated in RLT buffer prior to removal from the BSL-4 laboratory. Subsequently, RNA was isolated from blood using the QIAamp viral RNA kit (Qiagen), from tissues using the RNeasy minikit (Qiagen) according to the manufacturer’s instructions supplied with each kit.

### Hematology and serum biochemistry

As previously described^20^, blood was collected on days 0, 3, 6, and 10 p.i. and terminal endpoint for all animals. Total white blood cell counts, white blood cell differentials, red blood cell counts, platelet counts, hematocrit values, total hemoglobin concentrations, mean cell volumes, mean corpuscular volumes, and mean corpuscular hemoglobin concentrations were analyzed from blood collected in MiniCollect EDTA tubes (Greiner Bio One) using a Hemavet HV950FS instrument per manufacturer’s instructions (Drew Scientific, Oxford, CT). Serum was centrifuged at 2500 rpm for 10 minutes and analysis of blood chemistries was performed using a VetScan classic analyzer and comprehensive diagnostic profile rotors measuring of albumin, amylase, alanine aminotransferase, alkaline phosphatase, calcium, glucose, total protein, total bilirubin, blood urea nitrogen (BUN), creatinine, phosphorus, sodium, and total protein (Abaxis, Union City, CA). All blood and serum samples were processed and analyzed immediately after collection.

### Histopathology and immunohistochemistry

Necropsy was performed on all subjects as previously described^20^. Briefly, tissue samples of all major organs were collected for histopathologic and immunohistochemical examination and were immersion-fixed in 10% neutral buffered formalin for at least 21 days in BSL-4. Subsequently, formalin was changed; specimens were removed from BSL-4, processed in BSL-2 by conventional methods and embedded in paraffin and sectioned at 5 μm thickness as previously described^15^. Briefly, for immunohistochemistry, specific anti-NiV immunoreactivity was detected using an anti-NiV N protein rabbit primary antibody (kindly provided by Dr. Christopher Broder) at a 1:5000 dilution for 30 minutes. The tissue sections were processed for immunohistochemistry using the Dako Autostainer (Dako, Carpinteria, CA). Secondary antibody used was biotinylated goat anti-rabbit IgG (Vector Laboratories, Burlingame, CA) at 1:200 for 30 minutes followed by Dako LSAB2 streptavidin-HRP (Dako) for 15 minutes. Slides were developed with Dako DAB chromagen (Dako) for 5 minutes and counterstained with hematoxylin for one minute. Non-immune rabbit IgG was used as a negative staining control.

### Detection of rNiV_M_ load

As previously described^20^, RNA was isolated from blood or tissues and analyzed using primers/probe targeting the N gene and intergenic region between N and P of NiV for real-time quantitative PCR (RT-qPCR) with the probe used here being 6-carboxyfluorescein (6FAM)-5′ CGT CAC ACA TCA GCT CTG ACG A 3′-6 carboxytetramethylrhodamine (TAMRA; Life Technologies, Carlsbad, CA). This strategy using the intergenic region allows for genome and anti-genome detection only without detecting contaminating viral mRNA. rNiV_M_ RNA was detected using the CFX96 detection system (Bio-Rad) in One-step probe RT-qPCR kits (Qiagen) with the following cycle conditions: 50 °C for 10 minutes, 95 °C for 10 seconds, and 40 cycles of 95 °C for 10 seconds and 59 °C for 30 seconds. Threshold cycle (*CT*) values representing rNiV genomes were analyzed with CFX Manager Software, and data are shown as genome equivalents (GEq). To create the GEq standard, full-length NiV plamids were quantified using a Nanodrop 2000 (Thermo Scientific), and the number of NiV genomes was calculated using Avogadro’s number and the molecular weight of the full-length NiV plasmid containing the genome. Virus titration was performed by plaque assay with Vero cells from all blood and tissue samples. Briefly, increasing 10-fold dilutions of the samples were adsorbed to Vero cell monolayers in duplicate wells (200 μl); the limit of detection was 25 PFU/ml.

## Results

### Disrupting the STAT1-binding domain

**Figure 1:**
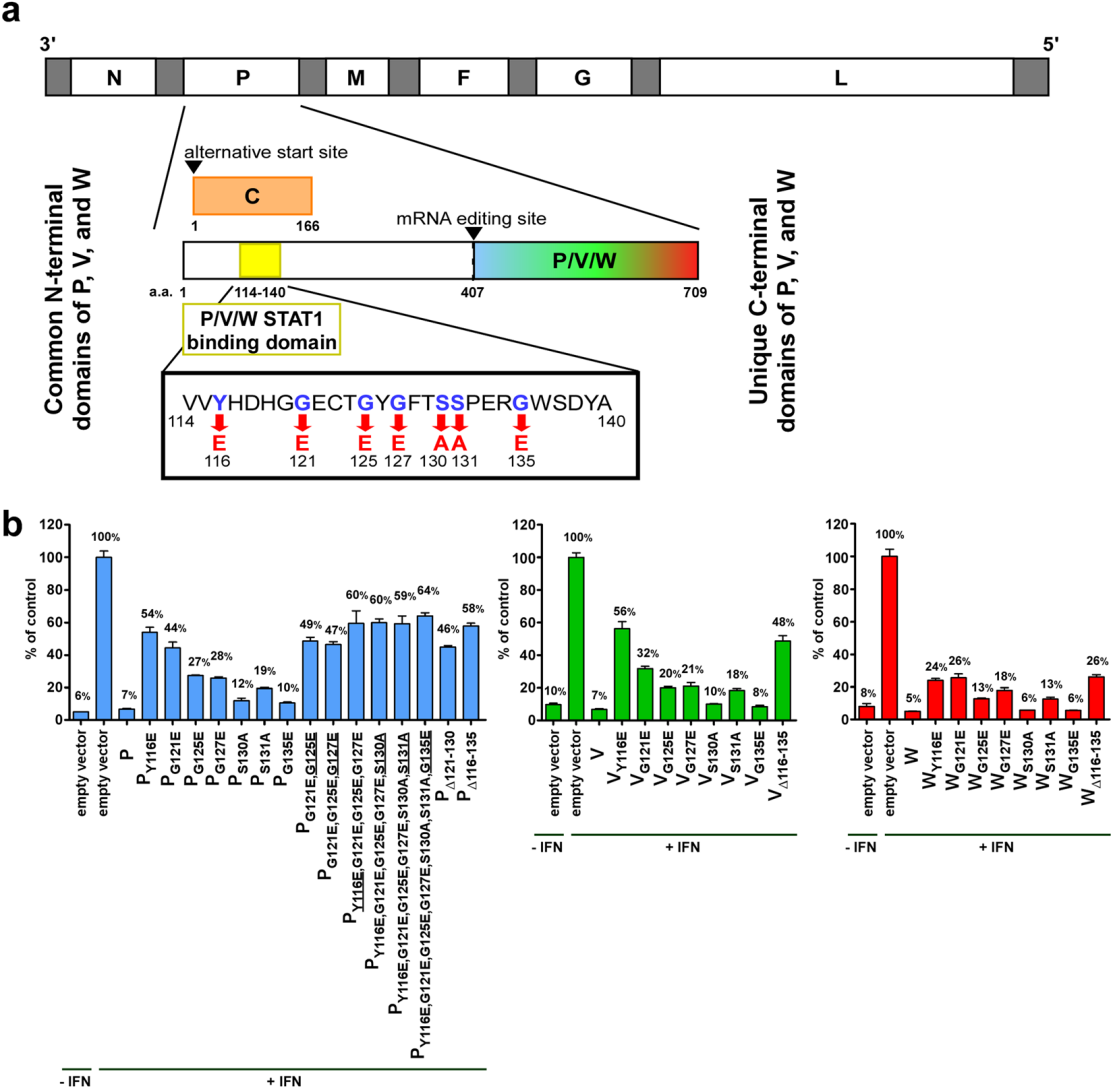
STAT1 binding domain and mutations. (**a**) Schematic of rNiV_M_ genome with the name of each gene indicated (N, P, M, F, G, and L). White segments indicate open reading frames and gray segments represent non-coding regions of the genome. Insert magnifies the P gene; the STAT1-binding domain of the NiV P, V, and W common N-terminus is located in amino acids 114-140 as indicated; this overlaps with the C protein ORF as shown. Seven amino acids involved in STAT1-binding (blue letters) disrupt STAT1-binding when mutated (red letters). The amino acid numbers flanking the domains are indicated. **(b)** Relative IFN-induced luciferase expression of 293 T cells transfected with various mutant pCAGGS-HA NiV_M_ P (blue), V (green), or W (red) expression plasmids after pre-treatment with universal IFN-α or non-treated control as indicated. Underlined mutations were added to the combination from the previous column.

The N-terminus of the NiV P protein is shared with the V and W proteins, and this region contains a STAT1-binding domain. Previous studies, using various methods, identified seven individual amino acid mutations located in this domain namely: Y116E, G121E, G127E, and G135E^24^, G125E^24,29^; and S130A and S131A^30^ (Fig. 1a). In order to directly compare these mutations using the same methods, these individual mutations, combinations of these mutations, and deletions of amino acids 121 to 130 (Δ121–130) or 116 to 135 (Δ116–135) were introduced into identical pCAGGS-HA NiV_M_ P expression plasmids. The individual mutations and the Δ116–135 deletion were also introduced into pCAGGS-HA NiV_M_ V and W expression plasmids.

Each plasmid was then transfected into 293 T cells along with firefly luciferase (driven by ISG54 promoter) and Renilla luciferase (driven by a constitutive CMV promoter) plasmids. Cells were treated with universal IFN-α then luciferase levels (both firefly and Renilla) were measured and the firefly luciferase:Renilla luciferase ratio was determined and used to calibrate for differential transfection efficiencies. The relative levels of ISG promoter activation as determined by this ratio are shown in Fig. 1b.

Several of the individual amino acid mutations purported to strongly prevent NiV P/V/W inhibition of ISG signaling demonstrated only a weak ability to prevent this inhibition in the present study. The Y116E and G121E mutations appeared to have the strongest ability to prevent this inhibition, although neither of these mutations were able to completely restore ISG signaling. The S130A, S131A, and G135E mutations demonstrated the least effect. Unlike similar studies in the measles virus (MeV) P/V STAT1-binding domain^47^, combining individual mutations did not lead to a marked, additive increase in the ability to prevent this inhibition. Instead, any combination examined simply had a similar ability to prevent this inhibition at a comparable level as that of the most potent single amino acid mutation included in the combination. Likewise, the Δ121–130 and Δ116–135 deletions were only able to prevent this inhibition to a similar level as the strongest single amino acid mutation included in the deletion (G121E and Y116E respectively).

Since no combination of mutations was able to completely restore ISG signaling, and to ensure that this reduction was not simply an effect of NiV_M_ protein synthesis, an additional experiment was performed including NiV_M_ N and L protein expression plasmids which achieved similar levels of ISG signaling as vector only while P again had strong suppression of ISG signaling, and P_Y116E_ and P_Δ116–135_ again restored partial ISG signaling (Supplementary Fig. 1).

### Co-immunoprecipitation and western blot analysis

**Figure 2:**
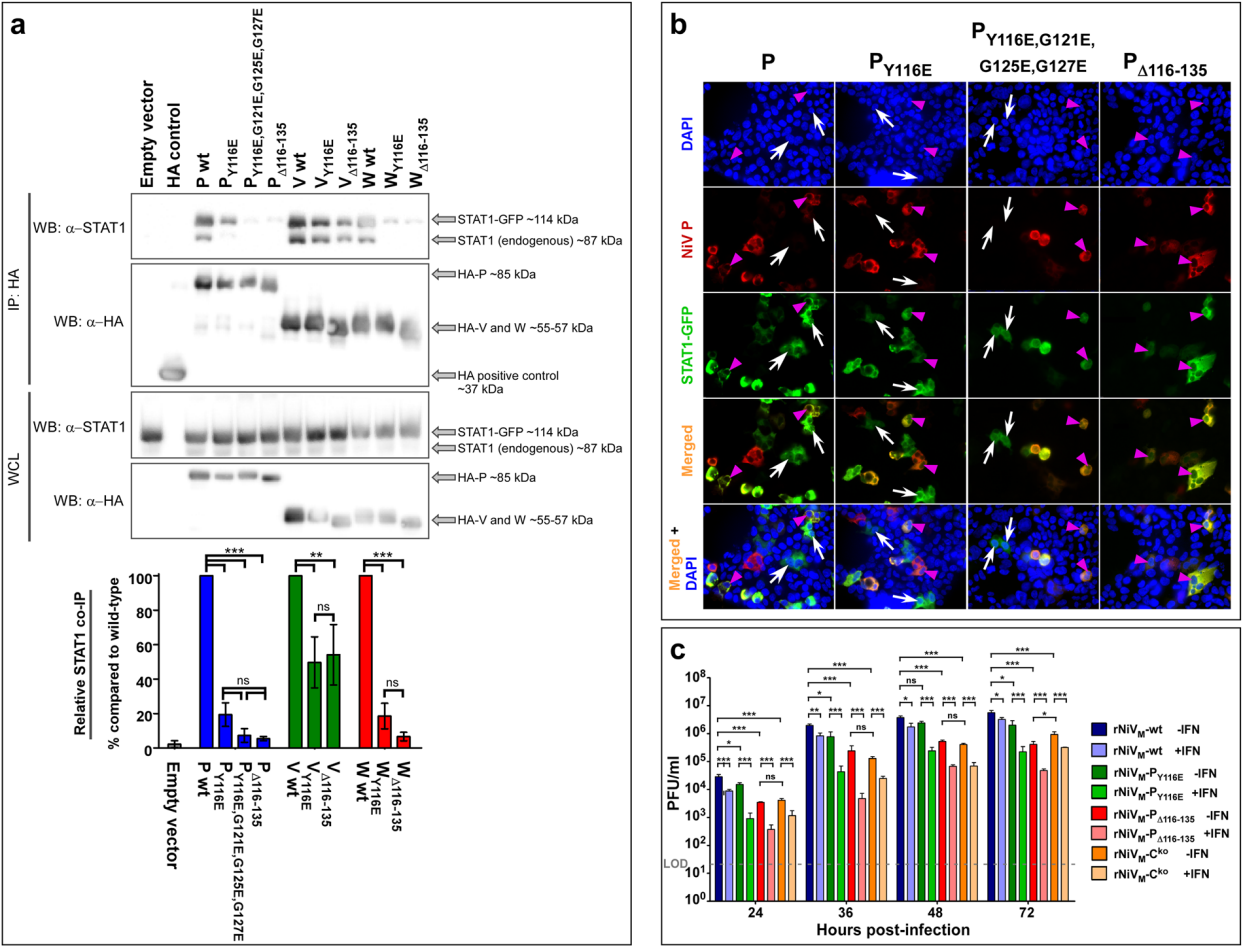
*In vitro* characterization of STAT1-binding mutations. (**a)** 293 T cells were transfected with pCAGGS-STAT1-GFP along with pCAGGS-HA NiV-P, -V, and -W wild-type and mutant proteins were then assessed at 24 hours post-transfection by Western blot, representative blots from three independent experiments are shown. Relative binding of P, V, and W mutants with STAT1 was assessed using co-immunoprecipitation. Amount of binding in the IP fraction was normalized to total STAT1 for the same well, then expressed as a ratio to the wild-type protein for each mutant as shown in the graph. Each cropped portion of Western blot is outlined in black to indicate separate blots stained with different antibodies as indicated (full Western blots of each is found in Supplementary Fig. 2). Differences between samples were assessed using one-way analysis of variance followed by Tukey’s post hoc test. **p-value < 0.01; ***p-value < 0.001; ns, not significant. N = 3; error bars represent standard error of the mean. IP: immunoprecipitation, WCL, whole cell lysate. **(b)** Transfected cells were treated with universal IFN-α then fixed. Cell nuclei are stained with DAPI (blue); cells transfected with HA-tagged NiV P protein (red) show P protein in the cytoplasm but not the nucleus; cells transfected with fused STAT1-GFP show STAT1 in both the cytoplasm and the nucleus when P protein is not co-transfected (white arrows). Cells transfected with STAT1-GFP show no STAT1 present in the nuclei when co-transfected with wild-type NiV P (magenta arrowhead; first column panels) but STAT1 is present when co-transfected with NiV P protein containing the P_Y116E_ mutation (magenta arrowhead; second column panels), P_Y116E,G121E,G125E,G127E_ mutations (magenta arrowhead; third column panels), or P_Δ116-135_ deletion (magenta arrowhead; fourth column panels) indicating the ability of these mutations to ablate the ability of NiV P to sequester STAT1. Images taken: 20×. **(c)** Growth kinetics in Vero cells either without pre-treatment of IFN-α (dark colors) or with pre-treatment of IFN-α (light colors). Error bars show standard deviation. LOD: Limit of detection of 25 PFU/ml. Analysis of variance (ANOVA) with Dunnett’s multiple comparison test; N = 4: *p-value < 0.05; **p-value < 0.01; ***p-value < 0.001.

To characterize the ability of the described mutations to affect the STAT1 binding, expression plasmids containing either wild-type or mutant P, V, and W proteins with HA tags were co-immunoprecipitated from cell lysates containing STAT1-GFP overexpression. The Y116E mutation was chosen as the single most potent mutant. The Δ116–135 mutation was chosen as it deletes the entire STAT1 binding region. The P_Y116E,G121E,G125E,G127E_ mutation was chosen as it contained the 4 most potent mutations. There was significant reduction in STAT1 that co-immunoprecipitated with Y116E and Δ116–135 mutations in P, V, and W, and P_Y116E,G121E,G125E,G127E_ (Fig. 2a; full Western blots seen in Supplementary Fig. 2).

### Immunofluorescence assays demonstrating STAT1 inhibition

To further demonstrate the ability of these mutations to prevent NiV P and V proteins from binding to and sequestering STAT1 within the cytoplasm, thus preventing nuclear translocation and subsequent ISG activation, transfection experiments with 293 T cells were conducted with the P or V expression plasmids containing wild-type sequence, the Y116E mutation, or the Δ116–135 deletion, together with a STAT1-GFP fusion expression plasmid. Stimulation of cells with universal IFN-α was performed and the subsequent immunofluorescence microscopy images for NiV P plasmids show STAT1 present in both the nucleus and cytoplasm of cells not expressing detectable amounts of NiV P (Fig. 2b, white arrows), but absent from the nucleus when co-expressed with NiV P wild-type (Fig. 2b, magenta arrowheads, first column); however, STAT1 is present in the nucleus in cells co-expressed with NiV P_Y116E_, NiV P_Y116E,G121E,G125E,G127E_, or NiV P_Δ116–135_ (Fig. 2b, magenta arrowheads, second, third, and forth columns, respectively) demonstrating that these mutations cannot sequester STAT1 in the cytoplasm as it does with wild-type P.

Similar results were seen for NiV V plasmids (Supplementary Fig. 3a). The same experiment was performed with the NiV W expression plasmids; however, since NiV W localizes to the nucleus, where it also sequesters STAT1 as previously described^37,41^, W was always found in the nucleus of the transfected cells (Supplementary Fig. 3b).

### Recombinant NiV production and *in vitro* characterization

rNiV_M_-wt^20^ and rNiV_M_-C^ko^ ^21^ were previously recovered and characterized while rNiV_M_-P_Y116E_ and rNiV_M_-P_Δ116–135_ mutants were recovered in the current study using similar reverse genetics methods. Growth kinetics were measured for each rNiV_M_ mutant in Vero cells (Fig. 2c dark colors). The previously described rNiV_M_-C^ko^ mutant is included in this study since the mutations in the STAT1-binding domain also produce mutations in the overlapping C protein ORF. A complete list of mutations used and their effects on the P and C ORFs is shown in Supplementary Fig. 4. The rNiV_M_-P_Y116E_ mutant had similar growth kinetics to rNiV_M_-wt and the growth kinetics were similar between the rNiV_M_-P_Δ116–135_ and rNiV_M_-C^ko^ mutants, although both of these were significantly different from the rNiV_M_-wt kinetics. These results bear similarity to previously reported rNiV_M_ mutants containing mutations in STAT1-binding and/or lacking C expression^21,24,25,27,32^. To examine the importance of the STAT1-binding domain in avoiding innate immunity, multi-cycle growth kinetics were measured in Vero cells that were pre-treated with IFN-α prior to infection (Fig. 2c light colors). All rNiVs grew to lower titers as seen previously^20,21^, however, the decrease was greater in the rNiV_M_-P_Y116E_ and rNiV_M_-P_Δ116–135_ mutants than in the rNiV_M_-wt or rNiV_M_-C^ko^ mutant, with the greatest difference being observed at 36 hours p.i.

### Disease and clinical observations in ferrets infected with the P_Y116E_ or P_Δ116–135_ mutants

**Figure 3:**
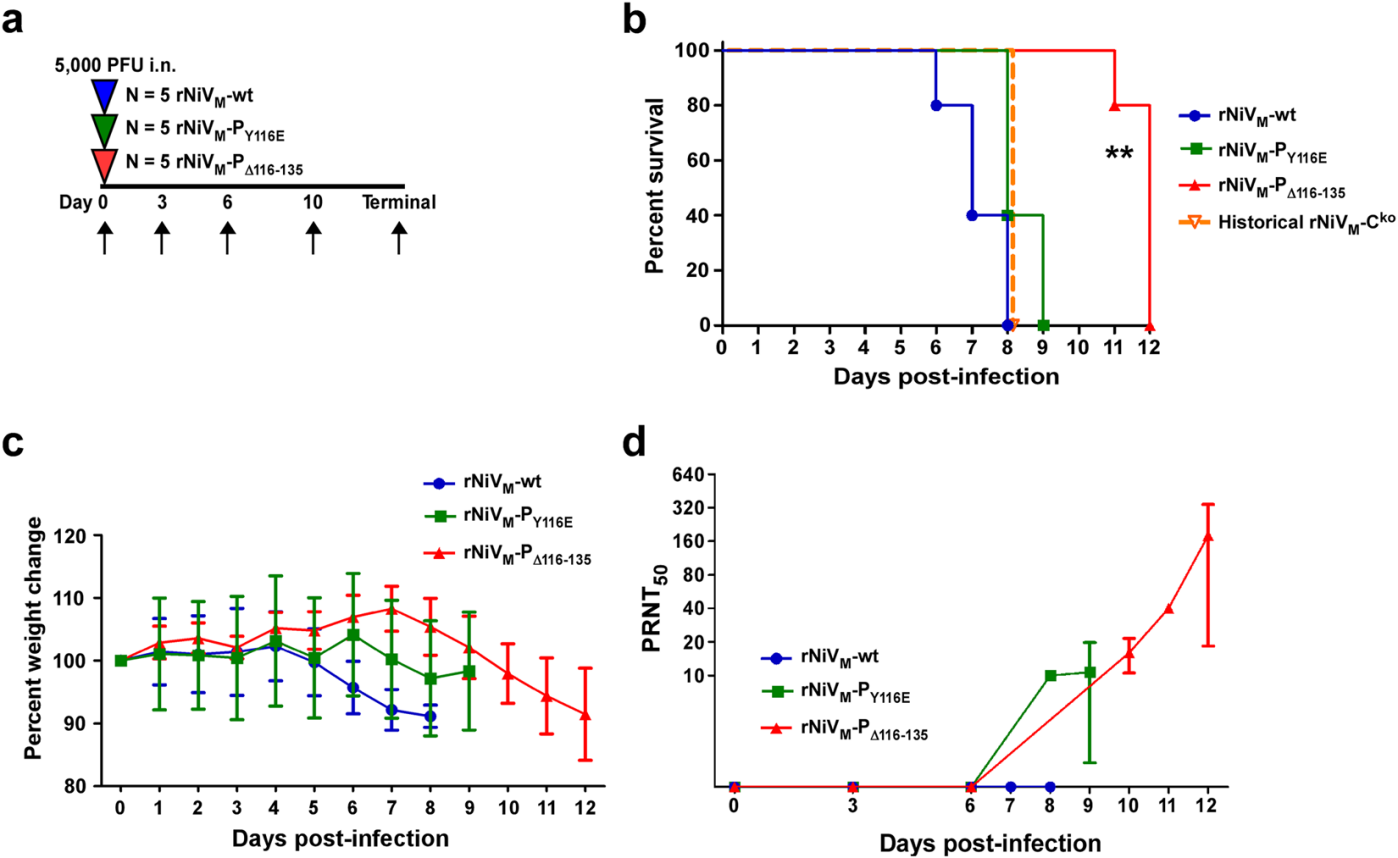
Clinical disease in ferrets after experimental infection with rNiV_M_. (**a)** Flow chart showing the day of infection (triangles) and days of samples collection (arrows). **(b)** Kapplan-Meier survival curve for ferrets infected with rNiV_M_-wt (blue circles), rNiV_M_-P_Y116E_ (green squares), rNiV_M_-P_Δ116-135_ (red triangles), or historical rNiV_M_-C^ko^ (open orange triangle and dotted line). Log-rank (Mantel–Cox) test; N = 5 for all rNiV_M_ ferret cohorts. **p-value < 0.01 compared to each of the rNiV_M_-wt, rNiV_M_-P_Y116E_, and rNiV_M_-C^ko^ curves. **(c)** Weight change for animals from the rNiV_M_-wt (blue circles), rNiV_M_-P_Y116E_ (green squares), and rNiV_M_-P_Δ116-135_ (red triangles) cohorts. Error bars show standard deviation. **(d)** Neutralizing antibody titers for animals from the rNiV_M_-wt (blue circles), rNiV_M_-P_Y116E_ (green squares), and rNiV_M_-P_Δ116-135_ (red triangles) cohorts. Error bars show standard deviation.

STAT1 is highly conserved among mammals; with 97% amino acid identity between humans and ferrets as shown in Supplementary Fig. 5. The ferret model has been used as a model of NiV pathogenesis and considering the homology of human and ferret STAT1, we chose to use the ferret model for our investigation of the NiV P/V/W STAT1 binding domain. Three groups of ferrets (n = 5 per group) were intranasally (i.n.) inoculated with 5 × 10^3^ PFU of virus (rNiV_M_-wt, rNiV_M_-P_Y116E_, or rNiV_M_-P_Δ116–135_). Temperature, weight, and clinical score were measured at least twice daily. Phlebotomy was performed on days 0, 3, 6, 10, and terminal days post-challenge with rNiVs (Fig. 3a).

The ferrets in the rNiV_M_-wt cohort uniformly succumbed between days 6–8 p.i. (Fig. 3b, blue). Signs of disease included fever, marked respiratory distress, and mild neurological disease (Table 1). The ferrets in the rNiV_M_-P_Y116E_ cohort uniformly succumbed between days 8–9 p.i. (Fig. 3b, green). Signs of disease included fever, mild to moderate respiratory distress, and neurological disease of variable severity (Table 1). The ferrets in the rNiV_M_-P_Δ116–135_ cohort succumbed between days 11–12 p.i. (Fig. 3b, red). Signs of disease included fever, the majority developed mild respiratory distress, and all developing severe neurological disease including limb paresis and/or seizures (Table 1). Since the mutations in the STAT1-binding domain also produce mutations in the overlapping C protein ORF, historical data for the rNiV_M_-C^ko^ cohort was included as a useful comparison, and this includes all animals succumbing on day 8 p.i. (Fig. 3b, orange dotted line). All animals showed weight loss before succumbing to disease (Fig. 3c).

**Table 1:** Clinical disease in ferrets after experimental infection with rNiV_M_.

Ferret no.	Clinical outcome	Resp*	Neuro^†^	Hem^‡^	Fever	Clinical disease
rNiV_M_-wt-1	EU d8	+	+	+	d4-7	Thrombocytopenia (d6,8); lymphopenia (d6); hypoalbuminemia (d6,8); >3-fold increase in BUN (d8); depression (d5-7); lethargy (d5-7); inappetence (d6-8); dehydration (d8); rales (d6-8); ocular, nasal, and oral discharge (d6); ataxia (d6-8); severe hypothermia (d8).
rNiV_M_-wt-2	EU d7	+	++	−	d4-6	Thrombocytopenia (d6,7); hypoalbuminemia (d7); hyperglycemia (d7); depression (d6-7); lethargy (d6-7); inappetence (d7); ocular and nasal discharge (d6-7); myoclonus (d7); ataxia (d7).
rNiV_M_-wt-3	EU d8	+	+	+	d4-6	Thrombocytopenia (d6,8); lymphopenia (d8); hypoalbuminemia (d6,8); >3-fold increase in BUN (d8); depression (d6-8); lethargy (d6-8); inappetence (d7-8); sneezing (d7-8); nasal discharge (d6-8); rales (d6-8); ataxia (d7-8); obtunded (d8); hypothermia (d8); loss of >10% body weight.
rNiV_M_-wt-4	EU d6	+	+	−	d5-6	Thrombocytopenia (d3,6); lymphopenia (d6); hypoalbuminemia (d6); depression (d6); lethargy (d6); nasal discharge (d6); myoclonus (d6); hypothermia (d6).
rNiV_M_-wt-5	EU d7	+	++	−	d4-6	Thrombocytopenia (d3,6,8); lymphopenia (d6); hypoalbuminemia (d6-7); >3-fold increase in BUN (d7); hyperglycemia (d6,7); depression (d6-7); lethargy (d6-7); inappetence (d6-7); ocular and nasal discharge (d7); rales (d7); myoclonus (d7); ataxia (d7); hindlimb paresis (d7); hypothermia (d7); loss of >10% body weight.
rNiV_M_-P_Y116E_-1	EU d8	+	+++	+	d5-7	Thrombocytopenia (d8); lymphopenia (d8); hypoalbuminemia (d6,8); hyperglycemia (d8); depression (d7-8); lethargy (d7-8); inappetence (d7-8); nasal discharge (d7-8); myoclonus (d8); seizures (d8); loss of >10% body weight.
rNiV_M_- P_Y116E_ -2	EU d8	+	+++	−	d6-8	Thrombocytopenia (d8); lymphopenia (d8); hypoalbuminemia (d8); >3-fold increase in BUN (d8); hyperglycemia (d8); depression (d7-8); lethargy (d7-8); oral and nasal discharge (d6-8); rales (d8); aggressive behavioral change with vocalization and visual deficit (d8); ataxia (d8); seizures (d8).
rNiV_M_- P_Y116E_ -3	EU d9	+	++	+	d7-9	Thrombocytopenia (d9); lymphopenia (d9); hypoalbuminemia (d9); hyperglycemia (d9); depression (d7-9); lethargy (d7-9); rales (d8-9).
rNiV_M_- P_Y116E_ -4	EU d9	+	+	+	d6-8	Thrombocytopenia (d9); lymphopenia (d9); hypoalbuminemia (d9); hyperglycemia (d9); depression (d7-9); lethargy (d8-9); nasal discharge (d6-9); rales (d8-9).
rNiV_M_- P_Y116E_ -5	EU d8	+	+++	+	d5-8	Thrombocytopenia (d8); lymphopenia (d6,8); hypoalbuminemia (d8); hyperglycemia (d8); depression (d7-8); lethargy (d7-8); nasal discharge (d6-8); hindlimb paresis (d8); seizures (d8).
rNiV_M_-P_Δ116-135_-1	EU d11	−	+++	+	d6-9	Thrombocytopenia (d6,10,11); lymphopenia (d6,10); >3-fold increase in BUN (d11); hyperglycemia (d8,11); depression (d6-8); lethargy (d6-8); inappetence (d10); left hindlimb paresis (d10); quadraparesis (d11); hypothermia (d12); loss of >10% body weight.
rNiV_M_-P_Δ116-135_-2	EU d12	+	+++	−	d6-11	Thrombocytopenia (d10,12); lymphopenia (d10); depression (d6-8); lethargy (d6-8); inappetence (9-10); sneezing (d9-10); nasal discharge (d10); facial and cervical tremors (d11-12); myoclonus (d11-12); hindlimb paresis (d12); hypothermia (d12); loss of >10% body weight.
rNiV_M_- P_Δ116-135_-3	EU d12	+	+++	−	d6-12	Thrombocytopenia (d12); lymphopenia (d6); depression (d6-8); lethargy (d6-8); inappetence (d9); seizures (d12).
rNiV_M_- P_Δ116-135_-4	EU d12	+	+++	+	d6-11	Thrombocytopenia (d10,12); lymphopenia (d6); hypoalbuminemia (d8); >3-fold increase in BUN (d12); hyperglycemia (d8); depression (d6-8); lethargy (d7-8); inappetence (d9); sneezing (d9-10); myoclonus (d10-12); quadraparesis (d12); severe hypothermia (d12); loss of >10% body weight.
rNiV_M_- P_Δ116-135_-5	EU d12	+	+++	−	d5-12	Thrombocytopenia (d10,12); lymphopenia (d12); hypoalbuminemia (d8,12); depression (d6-8); lethargy (d7-8); inappetance (d9-10); sneezing (d9); nasal discharge (d9); myoclonus (d11); hindlimb paresis (d12); loss of >10% body weight.

Resp, respiratory involvement; neuro, neurologic involvement; hem, hemorrhage; EU, euthanized due to rNiV_M_-mediated disease; d, day p.i.

*The absence **(−)** or presence **(**+**)** of increased respiratory effort and/or rate.

^†^The absence **(−)** or presence of minor **(**+**)**, moderate **(**++**)**, or severe **(**+++**)** neurological signs.

^‡^Extensive perioribtal, facial, and ventral neck edema with subcutaneous hemorrhages.

All blood samples were subjected to hematological and clinical chemistry analysis (Table 1). Regardless of cohort, as the infection progressed, clinical chemistry findings included > 3-fold increase in BUN without elevated creatinine (suggesting pre-renal azotemia, likely from dehydration), hypoalbuminemia, and hyperglycemia (suggesting pancreatic dysfunction and/or stress response); similarly, hematological findings in ferrets included lymphopenia and thrombocytopenia.

### Neutralizing antibody response

Humoral immunity was assessed by measuring serum neutralizing antibody with plaque reduction neutralization assay (PRNT). There were no detectable levels of neutralizing antibody in ferrets from the rNiV_M_-wt cohort while low amounts of neutralizing antibody were detected in most (4/5) ferrets in the rNiV_M_-P_Y116E_ cohort at the terminal bleeds on days 8/9 p.i. Similarly, low levels were observed in all ferrets from the rNiV_M_-P_Δ116–135_ cohort by day 10 p.i. which increased on terminal bleed days 11–12 p.i. (Fig. 3d).

### Gross pathology

**Figure 4:**
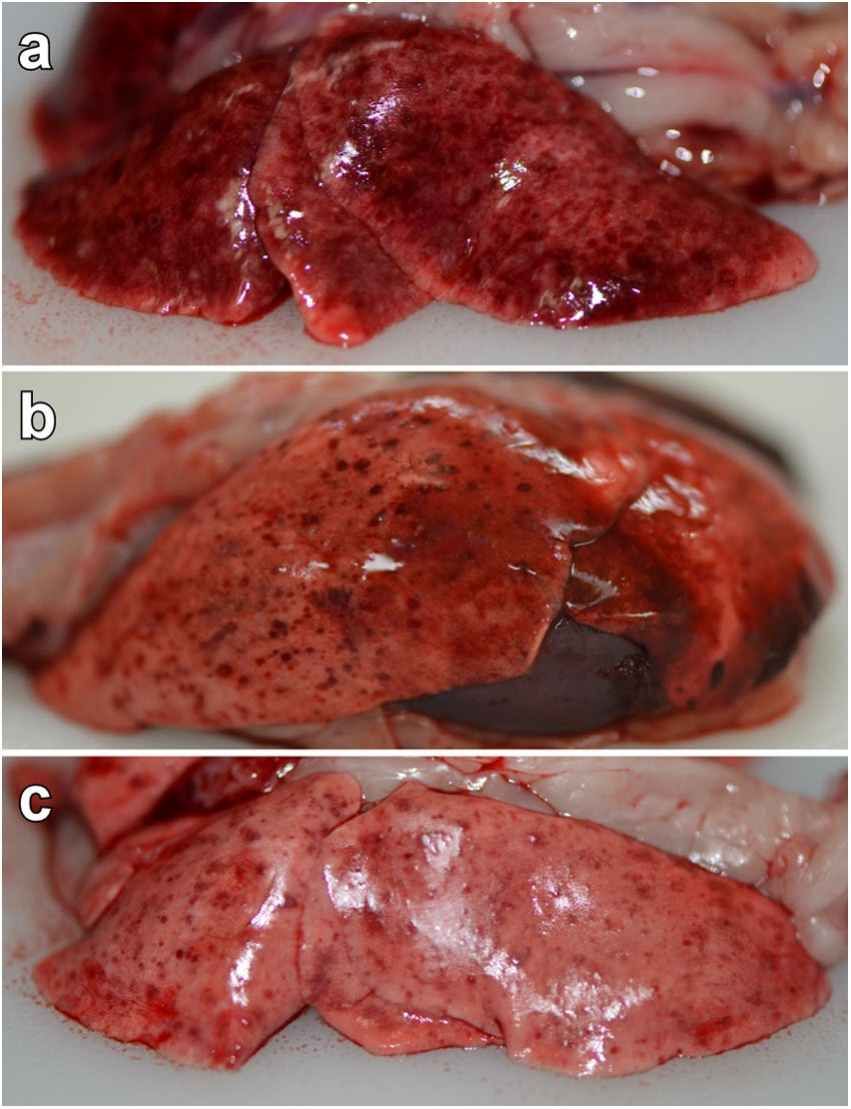
Gross pathology of lungs from rNiV_M_ infected ferrets. Representative gross pathology of lungs taken from ferrets infected with rNiV_M_-wt **(a)**, rNiV_M_-P_Y116E_ **(b)**, and rNiV_M_-P_Δ116-135_ **(c)**. Multifocal to coalescing hemorrhage and necrosis of all lung lobes is seen in rNiV_M_-wt infected ferrets **(a)**, while fewer and smaller numbers of hemorrhagic and necrotic foci are seen in rNiV_M_-P_Y116E_ infected ferrets **(b)**, and still fewer and smaller hemorrhagic foci are observed in rNiV_M_-P_Δ116-135_ infected ferrets **(c)**. Examples of normal ferret lungs can be seen in ref.^20^.

After NiV-disease mediated study endpoints all ferrets underwent necropsy. Marked differences were observed among the three cohorts with regards to the gross pathology of lung lesions with all ferrets (5/5) in the rNiV_M_-wt cohort demonstrating hemorrhagic and necrotizing pneumonia that was multifocal to coalescing (Fig. 4a). All ferrets (5/5) in the rNiV_M_-P_Y116E_ cohort demonstrated hemorrhagic and necrotizing pneumonia (Fig. 4b) consisting of multifocal pinpoint lesions of decreased severity compared to the rNiV_M_-wt cohort. All ferrets (5/5) in the rNiV_M_-P_Δ116–135_ cohort also had multifocal necrotizing pneumonia and pinpoint hemorrhages (Fig. 4c) which was markedly less severe than observed in the other cohorts; these were reminiscent of the less severe lesions observed in the previously published rNiV_M_-C^ko^ and rNiV_M_-W^ko^ cohorts^21^.

Mild blood vessel congestion within the meninges was observed in a minority of ferrets in the rNiV_M_-wt cohort (2/5), while marked congestion was observed in all animals from the rNiV_M_-P_Y116E_ (5/5) and the rNiV_M_-P_Δ116–135_ (5/5) cohorts (Supplementary Fig. 6a,b).

Splenic gross pathology was similar in ferrets from all three cohorts and consisted of splenomegaly with mottling and multifocal areas of pallor and dark erythema suggestive of necrosis as described previously^20^. Enlarged lymph nodes from the axial and inguinal chains were observed in some ferrets (Supplementary Fig. 6c,d). The kidneys from most animals in all three cohorts showed multifocal white and/or red foci on the surface of the kidneys indicating necrosis and hemorrhagic infarcts, respectively (Supplementary Fig. 6e). Areas of mucosal hemorrhage were observed in the urinary bladder from many ferrets from all three cohorts (Supplementary Fig. 6f).

### Histopathology and immunohistochemistry

**Figure 5:**
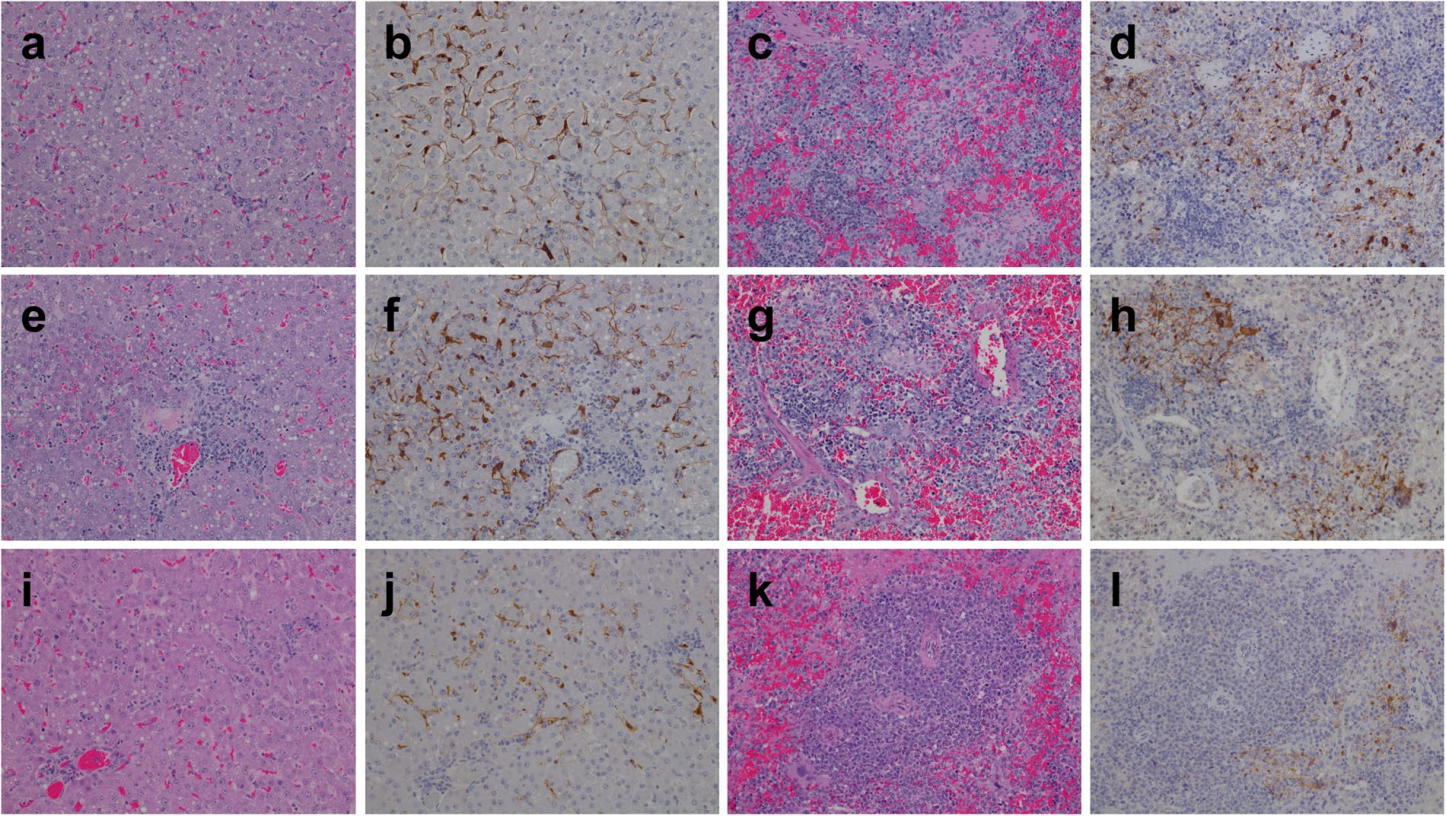
H&E and immunohistochemistry of ferret liver and spleen. Representative H&E (**a**,**c**,**e**,**g**,**i**,**k**) and immunohistochemistry labeled with a NiV N protein-specific polyclonal rabbit antibody (**b**,**d**,**f**,**h**,**j**,**l**). Liver (**a**,**b**,**e**,**f**,**i**,**j**) and spleen (**c**,**d**,**g**,**h**,**k**,**l**) from representative ferrets infected with rNiV_M_-wt (**a**–**d**), rNiV_M_-PY116E (**e**–**h**), and rNiV_M_-PΔ116-135 (**i**–**l**). Images taken: liver 20×, spleen 20×. Normal ferret histology for these tissues can be seen in ref.^20^.

Hematoxylin and eosin (H&E) staining was performed on various tissue specimens as was immunohistochemistry (IHC) using NiV N protein specific antibodies. Histopathology demonstrated liver lesions in all ferrets in the rNiV_M_-wt (Fig. 5a), rNiV_M_-P_Y116E_ (Fig. 5e), and rNiV_M_-P_Δ116–135_ (Fig. 5i) cohorts which included moderate congestion, mild to moderate vacuolar change, hepatocellular necrosis, moderate to severe periportal lymphoplasmacytic infiltrates, and occasional sinusoidal neutrophilic leukocytosis. Strong NiV immunolabeling was seen in all ferrets from the rNiV_M_-wt (Fig. 5b) and rNiV_M_-P_Y116E_ (Fig. 5f) with weaker immunolabeling in the rNiV_M_-P_Δ116–135_ cohort (Fig. 5j). Additionally, there were multifocal areas of immunolabeling observed in the cells lining the sinusoids, sinusoidal mononuclear (Kupffer) cells, endothelial cells of the surrounding medium to large caliber blood vessels, and within occasional mononuclear cells observed around necrotic areas.

All ferrets in the rNiV_M_-wt cohort (Fig. 5c) had splenic lesions which demonstrated syncytial cells, moderate to marked lymphoid follicle depletion, hemorrhage, and deposits of fibrin within the white pulp that included cellular debris together with both viable and degenerative neutrophils. All ferrets in the rNiV_M_-wt cohort (Fig. 5d) showed strong immunolabeling in syncytial cells, occasional endothelial cells, and mononuclear cells found within remnants of lymphoid follicles. In contrast, spleens from ferrets in the rNiV_M_-P_Y116E_ cohort (Fig. 5g) and rNiV_M_-P_Δ116–135_ cohort (Fig. 5k) demonstrated only mildly disturbed architecture with hypercellularity of the red pulp, hemorrhage, and occasional necrotic areas with little germinal center disorganization, although these occasional areas were associated with the presence of moderately strong (rNiV_M_-P_Y116E_; Fig. 5h) or minimal (rNiV_M_-P_Δ116–135_; Fig. 5l) immunolabeling of scattered mononuclear cells and endothelium.. Of note, the splenic architecture seen in the previously described rNiV_M_-C^ko^ cohort^21^ had a similar appearance to the rNiV_M_-wt cohort described here and this is in stark contrast with the mostly intact architecture seen in the rNiV_M_-P_Y116E_ and rNiV_M_-P_Δ116–135_ cohorts.

**Figure 6:**
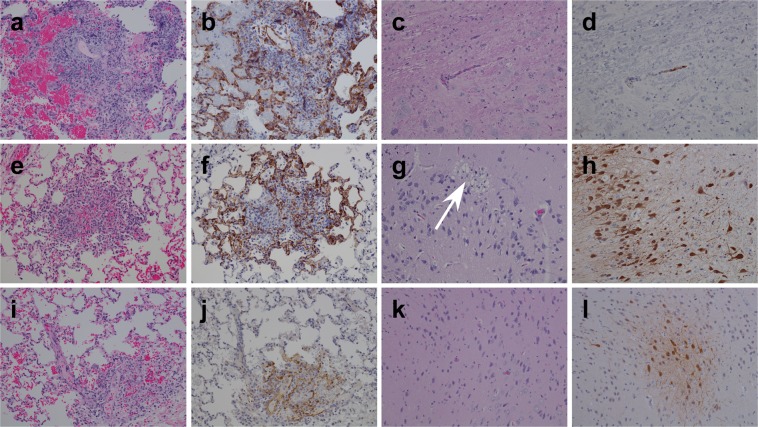
H&E and immunohistochemistry of ferret lung and brain. Representative H&E **(a**,**c**,**e**,**g**,**i**,**k)** and immunohistochemistry labeled with a NiV N protein-specific polyclonal rabbit antibody **(b**,**d**,**f**,**h**,**j**,**l)**. Lung **(a**,**b**,**e**,**f**,**i**,**j)** and brain **(c**,**d**,**g**,**h**,**k**,**l)** from representative ferrets infected with rNiV_M_-wt **(a**–**d)**, rNiV_M_-P_Y116E_ **(e**–**h)**, and rNiV_M_-P_Δ116-135_ **(i**–**l)**. White arrow points to area of marked locally extensive, vacuolar plaque with necrosis and gliosis within the grey matter in regions with diffuse, strongly immunopositive neuronal involvement. No vacuolar plaque is observed without associated NiV antigen in the frontal cortex of rNiV_M_-P_Y116E_-02. Images taken: lung 20×, brain 20×. Normal ferret histology for these tissues can be seen in ref.^20^.

Lesions were seen in lungs from ferrets in the rNiV_M_-wt (Fig. 6a), the rNiV_M_-P_Y116E_ (Fig. 6e), and the rNiV_M_-P_Δ116–135_ (Fig. 6i) cohorts which included various amounts of alveolar septae necrosis, nodular inflammation, and interstitial pneumonia. Scattered syncytial cell formation was also observed among endothelial cells and respiratory epithelial cells. The rNiV_M_-wt cohort (Fig. 6b) showed strong NiV immunolabeling within respiratory epithelial, mononuclear, and endothelial cells. The rNiV_M_-P_Y116E_ (Fig. 6f) and rNiV_M_-P_Δ116–135_ (Fig. 6j) cohorts demonstrated immunolabeling (strong and weak, respectively) that was primarily localized to the small areas of nodular inflammation. There was also considerably less edema and hemorrhage in these cohorts than in the rNiV_M_-wt cohort.

Routine brain section H&E staining was without lesions for all ferrets in the rNiV_M_-wt (Fig. 6c) and rNiV_M_-P_Δ116–135_ (Fig. 6k) cohorts. Only one animal, rNiV_M_-P_Y116E_-02, in the rNiV_M_-P_Y116E_ cohort exhibited any detectable lesion in the brain on H&E sectioning (Fig. 6g) which showed marked locally extensive, vacuolar plaque with necrosis and gliosis within the grey matter. Intense NiV antigen immunostaining was observed in small vessel endothelial cells within the brainstem, cerebrum, meninges, and choroid plexus in most (4/5) ferrets from the rNiV_M_-wt (Fig. 6d) cohort. Individual animals in the rNiV_M_-P_Y116E_ cohort had a significant difference in lesion severity associated with the brain. Ferrets rNiV_M_-P_Y116E_-01 and rNiV_M_-P_Y116E_-05 had extensive immunolabeling of granular cells within the cerebellum but no cerebral lesions (Supplementary Fig. 7a,b). Ferret rNiV_M_-P_Y116E_-02 displayed striking lesions with extensive neuronal immunolabeling in the cerebrum, this included the region with the vacuolar plaque seen on H&E (Fig. 6g,h; Supplementary Fig. 7c,d). Immunolabeling was detected in small vessel endothelium in the remaining two ferrets, but no neuronal immunolabeling was present in these ferrets. All ferrets in the rNiV_M_-P_Δ116–135_ cohort had multiple areas with intense NiV antigen immunolabeling within endothelial and neuronal cells in multiple areas of the brain including the brainstem, cerebrum, cerebellum, and/or hippocampus (Fig. 6l). Meningitis was occasionally seen in ferrets from the rNiV_M_-wt (Supplementary Fig. 8a,b) or rNiV_M_-P_Y116E_ (Supplementary Fig. 8e,f) cohorts; however, widespread meningitis was observed in the brains of all animals in the rNiV_M_-P_Δ116–135_ cohort (Supplementary Fig. 8i,j).

Renal histopathologic lesions from all ferrets in all three cohorts (Supplementary Fig. 8c,g,k) included inflammatory cell infiltration, glomerulonephritis, sclerotic glomeruli, tubular necrosis with vacuolar degeneration, and the presence of hyaline casts and granular casts. NiV antigen immunostaining in the rNiV_M_-wt (Supplementary Fig. 8d), rNiV_M_-P_Y116E_ (Supplementary Fig. 8h), and rNiV_M_-P_Δ116–135_ (Supplementary Fig. 8i) cohorts was detected in sporadic mononuclear cells, glomerular endothelium, other vascular endothelium, and renal tubular epithelium.

### Viral load

**Figure 7:**
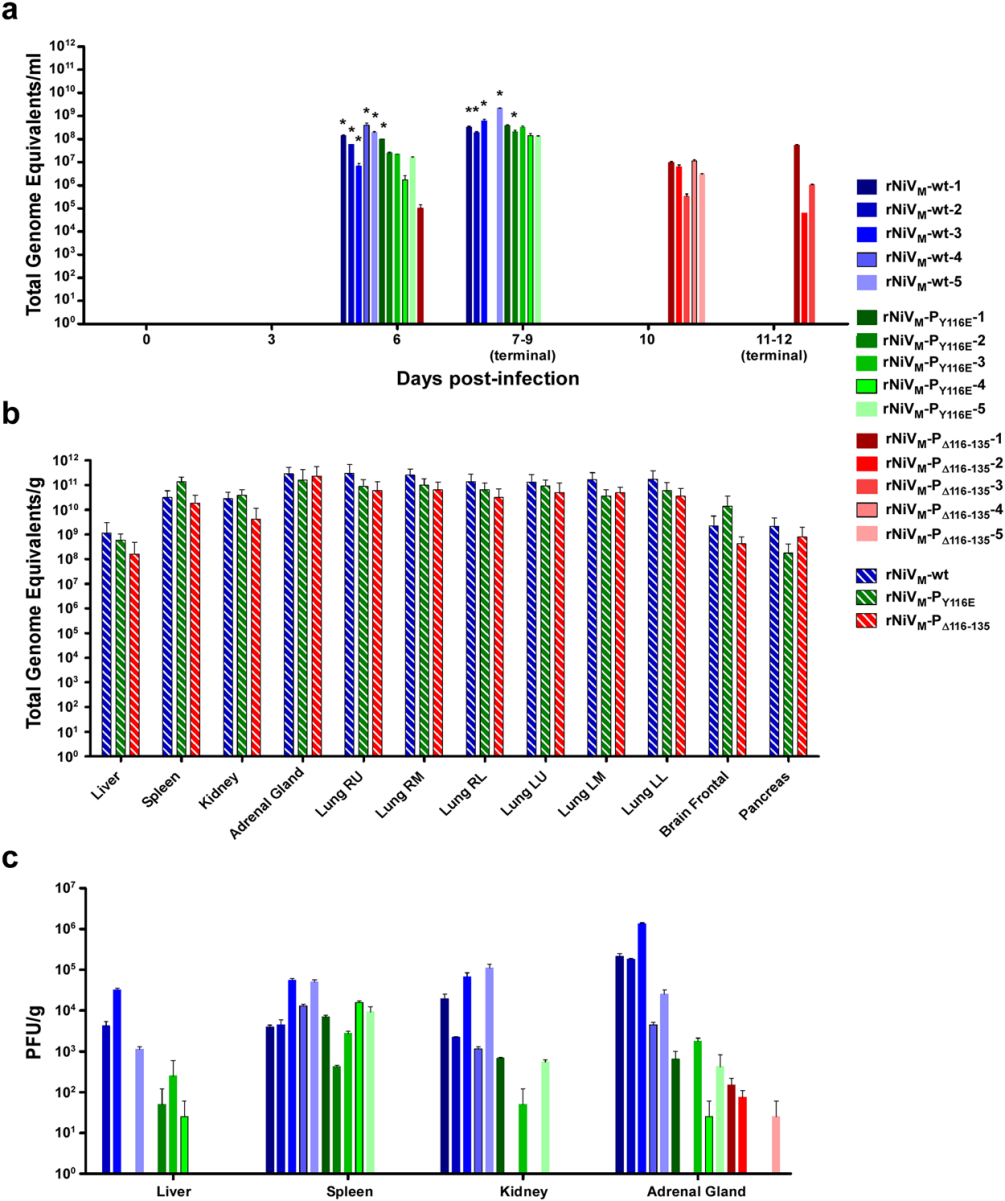
Viral load in rNiV_M_ infected ferrets. Viral load in ferrets as detected by genome equivalents (GEq) by qRT-PCR from (**a**) blood as GEq/ml and from (**b**) tissues as GEq/g (**c**) PFU/g of rNiV_M_ isolated from tissues of ferrets after necropsy. Hashed lines represent the average of all animals in the respective cohorts. Error bars show standard deviation. N = 5 for hashed lines; N = 2 for all other lines. Right upper (R.U.), right middle (R.M.), right lower (R.L.), left upper (L.U.), left middle (L.M.), left upper (L.U.). *Indicates samples where rNiV_M_ was successfully isolated from whole blood samples.

RT-qPCR was performed and quantified the level of viral genome from blood samples (Fig. 7a). All animals infected with rNiV_M_-wt or rNiV_M_-P_Y116E_ had measurable viral genome on day 6 p.i. which persisted through the disease course. Notably, viral genome could only be detected in the blood of a single animal infected with rNiV_M_-P_Δ116–135_ on day 6 p.i., however all 5 had detectable levels by day 10 p.i. Virus isolation was attempted for all blood samples, but was only successful for the samples from all 5 of the rNiV_M_-wt cohort animals and from 2 samples (one from day 6 and the other from day 8 p.i.) from 2 different rNiV_M_-P_Y116E_ cohort animals; no isolation was successful from any rNiV_M_-P_Δ116–135_ cohort blood samples.

Viral loads from multiple tissues were determined by using RT-qPCR on RNA extracted from tissue homogenates (Fig. 7b) with NiV genome present in all ferrets in all three cohorts. NiV was isolated from liver, spleen, kidney, and adrenal gland samples in all ferrets from the rNiV_M_-wt cohort with the exception of a few liver samples; similarly, virus was isolated from most rNiV_M_-P_Y116E_ cohort samples, with the exceptions being 2 liver, 2 kidney, and one adrenal gland samples; however, virus was only able to be isolated from 3 rNiV_M_-P_Δ116–135_ cohort adrenal gland samples and not from any liver, spleen, or kidney samples (Fig. 7c).

## Discussion

Although previous over-expression studies have identified amino acids of importance to the NiV STAT1-binding domain shared by the P, V, and W proteins^24,29,30^, when we used one set of standardized, well controlled methods, it became obvious that the seven previously described mutations purported to disrupt STAT1 binding and IFN signaling were not all of equal importance, in fact, some of the mutations appeared to have a less than anticipated effect on IFN signaling (Fig. 1b) or binding (Fig. 2a). It was also surprising that combinations of these mutations did not appear to have an additive effect as did the combination of similar mutations in MeV^47^. However, despite this, both the Y116E and Δ116–135 mutations were able to functionally prevent the NiV P and V proteins from cytoplasmically sequestering STAT1 at levels that appeared comparable to cells with no P or V present (Fig. 2b, Supplementary Fig. 3a). Altogether, these data suggest that both the rNiV_M_-P_Y116E_ and rNiV_M_-P_Δ116–135_ mutants can be considered STAT1^blind^ similar to those described for related MeV^47^ and canine distemper virus (CDV)^48^.

The P_Δ116–135_ mutation used in this study not only deletes 20 amino acids from the STAT1-binding domain, but also deletes 20 amino acids from the 166-amino acid long C protein as these ORFs overlap (Fig. 1a; Supplementary Fig. 4). This deletion may disrupt the function of the C protein since rNiV_M_-P_Δ116–135_ grows to similarly low titers in cell culture as rNiV_M_-C^ko^. The rNiV_M_-P_Y116E_ mutant appears to produce a truncated C protein (Supplementary Fig. 4) and it grows to slightly lower titers than rNiV_M_-wt but not as low as rNiV_M_-C^ko^ or rNiV_M_-P_Δ116–135_. This is in contrast to a G121E mutation in the STAT1-binding domain published previously^32^ which was able to grow to similarly high titers as rNiV_M_-wt. It is notable that both rNiV_M_-P_Y116E_ and rNiV_M_-P_Δ116–135_ were more susceptible to IFN-α treatment than rNiV_M_-wt or rNiV_M_-C^ko^ (Fig. 2c).

The previously published rNiV_M_-C^ko^ cohort had similar amounts of NiV genomes in blood and infectious virus in tissues as the rNiV_M_-wt cohort, and the rNiV_M_-P_Y116E_ in the present study had only slightly lower levels, while the rNiV_M_-P_Δ116–135_ cohort had markedly lower levels (Fig. 7). In addition, the rNiV_M_-C^ko^ cohort all succumbed within the same timeframe (day 8 p.i.) as the rNiV_M_-wt cohort (days 6–8 p.i.), and the rNiV_M_-P_Y116E_ cohort was only slightly delayed without statistical significance (days 8–9 p.i.), while the rNiV_M_-P_Δ116–135_ cohort succumbed at statistically significant delayed time points (days 11–12 p.i.; Fig. 3b). Therefore, it appears likely that there is a synergistic effect between the probable defect in the C protein and the loss of the STAT1-binding domain reminiscent of the synergistic effect observed with the combined mutant rNiV_M_-C^ko^W^ko^ previously published^21^. Indeed, a triple mutant including the P_Y116E_ mutation with the C^ko^W^ko^ mutations may synergistically lead to even further attenuation and would prove an enlightening counterpoint to studies involving knocking out MDA5/RIG-I or STAT2 binding since these are the remaining known targets of the NiV P gene products not yet explored. It is also possible that the deletion of the STAT1-binding domain may have other effects not directly related to STAT1.

STAT1 binding appears to be a major determinant of virulence in MeV^47^, but not so in CDV^48^, which is a frequently used surrogate for MeV due to the fact that MeV is non-lethal in animal models (usually rhesus macaques) while CDV is lethal in the ferret model. One study found that an rMeV containing mutations preventing the ability to bind STAT1 (STAT1^blind^), produced attenuated disease in rhesus macaques with lower titers in PBMCs and lymphoid tissue, having more rapid viral clearance, and demonstrating decreased clinical pathogenicity^47^. However, a similar STAT1^blind^ rCDV retained full pathogenicity and all animals developed lethal disease, while rCDV mutants with defective STAT2- or MDA5-binding domains were attenuated and all animals survived after only a mild disease^48^. It is not clear if the differences observed in the STAT1^blind^ rMeV and rCDV mutants are due to the difference in the viruses, their tropism, or in the host species response, rhesus macaques and ferrets, respectively.

Similar to the rCDV-STAT1^blind^ discussed above, the rNiV_M_-P_Y116E_ and rNiV_M_-P_Δ116–135_ still caused lethal disease in ferrets. The deletion of the STAT1-binding domain however did lead to an altered disease course with less extensive lesions in the lungs, liver, spleen, and kidneys. There was greater pathology observed in the brains of the rNiV_M_-P_Y116E_ and the rNiV_M_-P_Δ116–135_ cohorts however, including congested meningeal blood vessels, more frequent meningitis, and extensive neuroinvasion with antigen being found in neurons from multiple brain regions (hippocampus, brainstem, cerebellum, and cerebrum). This corresponded with severe neurological signs including seizures and occasional hindlimb paresis in the rNiV_M_-P_Y116E_ cohort and seizures with paralysis that progressed in a consistent manner beginning with one hindlimb, then both hindlimbs, and finally the forelimbs at which point the animals met study endpoint criteria of disease in a similar manner to the ferrets which succumbed in the previously described rNiV_M_-C^ko^W^ko^ cohort^21^, albeit in a more narrow timeframe. In fact, the level of clinical neuroinvasion observed in the rNiV_M_-P_Δ116–135_ cohort (Table 1) appeared to be greater and more consistent than that observed in any of our previously described cohorts including the rNiV_M_-C^ko^, rNiV_M_-W^ko^, or rNiV_M_-C^ko^W^ko^ ^20,21^ cohorts or the rNiV_M_-P_Y116E_ cohort from the present study, all of these cohorts, however, demonstrated greater neuroinvasion than seen in rNiV_M_-wt infection.

The minimal splenic architectural alterations, including hypercellularity of the red pulp, of the rNiV_M_-P_Y116E_ cohort, and even more so in the rNiV_M_-P_Δ116–135_ cohort (Fig. 5), were similar to that observed in the ferrets that succumbed to rNiV_M_-C^ko^W^ko^ ^21^. Each of these cohorts was still capable of generating neutralizing antibody as opposed to the rNiV_M_-wt, rNiV_M_-C^ko^, and rNiV_M_-W^ko^ cohorts, which had extensive germinal center necrosis and could not produce neutralizing antibody responses, even as far out as day 11 p.i. Also, similar to the rNiV_M_-C^ko^W^ko^ cohort, the neutralizing antibody response of the rNiV_M_-P_Δ116–135_ cohort may have helped reduce the amount of viral genome (Fig. 7) and antigen (Figs 5 and 6) observed in many organ systems, it did not prevent NiV from invading the CNS and leading to lethal neurological disease. This is perhaps due to the fact that neutralizing antibody is not detected in these animals until after day 6 p.i. (Fig. 3d) when NiV has already begun invading the CNS, and antibodies do not readily cross the blood brain barrier (BBB).

There are several possible explanations for the altered disease course observed with the STAT1^blind^ mutations in rNiV_M_. One possibility is that the severe neurological disease observed is simply the natural progression of NiV disease if animals are able to survive the respiratory disease and live long enough for the neurological disease to progress. The fact that some ferrets in the rNiV_M_-P_Y116E_ cohort showed markedly advanced neuronal infection on IHC compared to rNiV_M_-wt ferrets which succumbed at similar days indicates that this cannot be the only explanation. Therefore, it is probable that when NiV is unable to control the cellular cytokine responses due to lack of immunomodulatory proteins or domains, that small foci of NiV-infected endothelial cells may lead to BBB micro-breaches permitting occasional entry of virus without coincident entry of inflammatory cytokines and antibodies^49,50^ and the possible connection with NiV infection was discussed in more detail previously^21^.

Another possible explanation may be more directly related to Jak/STAT signaling. It has been shown that different cell types respond differently to cytokine stimulation^51,52^ with evidence suggesting that Jak/STAT signaling through type I IFN not only has an antiviral role in neurons^53^, but that it is also involved in deleterious neuro-inflammatory events^54^ and neurodegeneration^55^ in various non-viral diseases and is not always activated in the same way as in non-neuronal cells^52,56^. Therefore, it is possible that the ability of NiV to inhibit STAT1 may be more important in various non-neuronal cells but be less important in neurons, leading to the decreased viral spread through various organ systems, but not hindering spread through the CNS, particularly in the NiV_M_-P_Δ116–135_ cohort. Indeed, there is some evidence that this is occurring in the rare instances of MeV infection of the CNS^56^ where STAT1 binding plays a major role in most cell types, but neurons use a STAT1-independent mechanism of IFN signaling to clear MeV infection in mice, suggesting that STAT1 binding is less important in neurons than other cell types. Additional studies are warranted to better determine the mechanisms and temporal relationships as NiV infection progresses through the various organs including CNS invasion through more extensive, serial sacrifice experiments. This would allow for a better understanding of what role the various NiV proteins play in altering the temporal spread of NiV through the respiratory, lymphoid, CNS, and other organ systems. Additionally, it is possible that the initial cells of major virus replication might be different in the rNiV_M_ mutants, thus contributing to the different invasion patterns observed.

**Figure 8:**
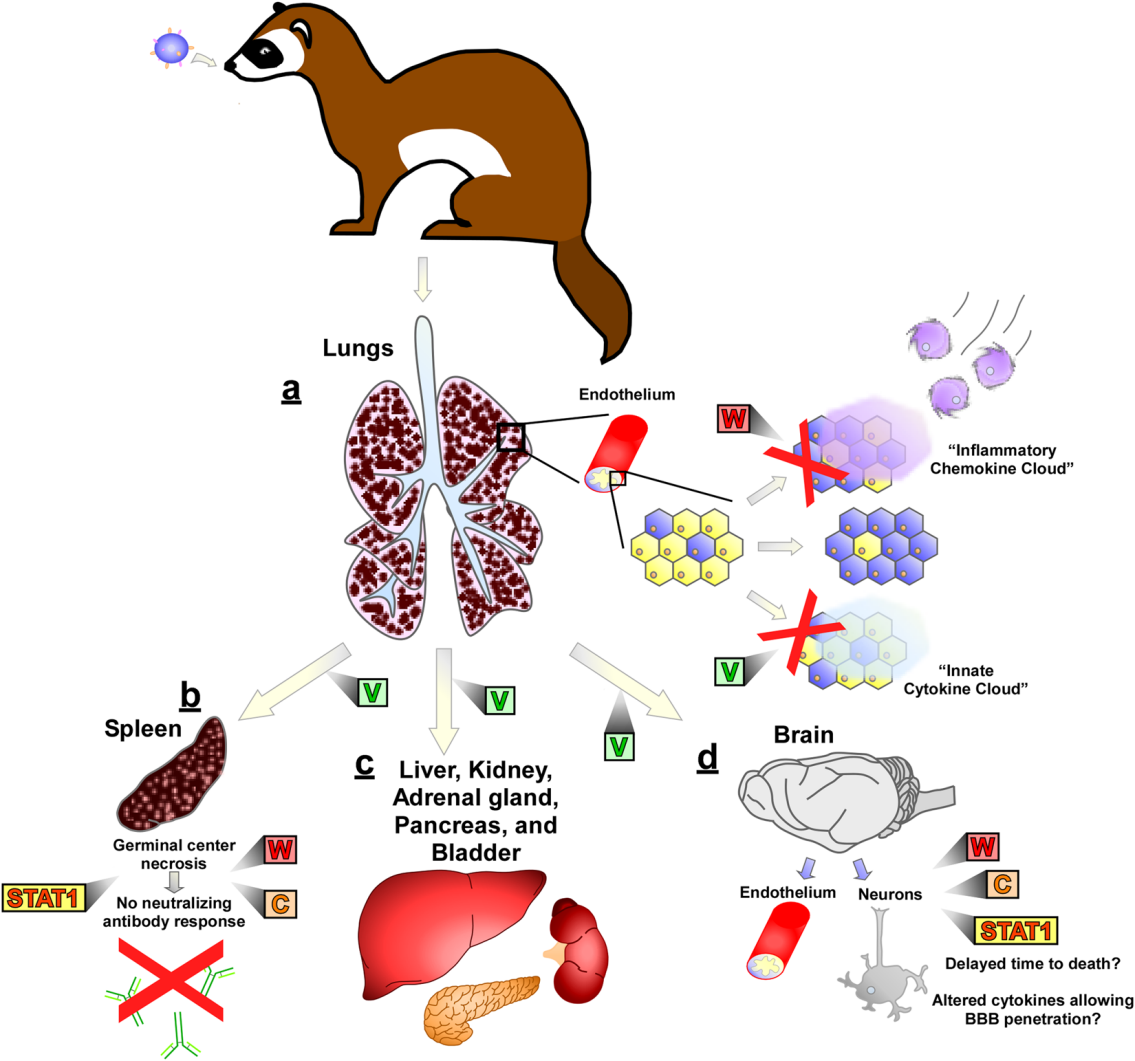
Contributions of the NiV P gene products to pathogenesis in the ferret model. (**a)** After i.n. challenge of ferrets with NiV, early targets include lung endothelial cells. The V protein prevents induction of an “innate cytokine cloud” that would inhibit spread of NiV through the endothelium. The W protein prevents induction of an “inflammatory chemokine cloud” that would recruit leukocytes to control NiV-mediated pulmonary lesions. After spreading through lung endothelium, NiV rapidly spreads to multiple organ systems. **(b)** Spread to the spleen leads to germinal center necrosis and a subsequent lack of neutralizing antibody production. This ability of NiV to prevent neutralizing antibody production is mediated by multiple factors: NiV must efficiently spread to the spleen, for which the V protein is essential; additionally, both the ability of P/V/W to inhibit STAT1 and expression of either the W or C protein must be functional to allow for germinal center destruction and prevention of a neutralizing antibody response. **(c)** Endothelial and parenchymal cells of multiple other organ systems, including liver, kidney, adrenal gland, pancreas, and urinary bladder also become infected. This often leads to blood chemistry abnormalities including increases in BUN and glucose levels. **(d)** Eventually NiV spreads to the CNS with infection of the endothelium with mild neurological signs, although the ferrets succumb before many neurons become infected, more extensive infection of neurons, together with more extensive neurological clinical signs, can be observed when either the W and/or C proteins are not expressed, or when NiV is unable to inhibit STAT1. This may be due to increased time to death and/or to altered cytokine expression by infected cells allowing penetration of the blood brain barrier.

In summary, the rNiV_M_ ferret experiments presented above together with our previous studies^20,21^ demonstrate that disruption of the NiV V, W, or C proteins as well as the P/V/W STAT1-binding domain lead to altered signs of clinical disease (Table 2) and altered pathological findings (Supplementary Table 1) when compared with rNiV_M_-wt infection in ferrets providing further evidence that the major pathogenic determinant is the V protein. STAT1 antagonism through the NiV P protein products plays only a minor role in neuropathogenesis in ferrets and is not necessary for neuroinvasion. The current role of the NiV P, V, W, and C proteins in the ferret model are summarized in Fig. 8. Future work should further elucidate the pathways in innate immunity important to NiV infection and confirming probable targets of importance for NiV proteins including MDA5 and STAT2 as well as identifying novel targets.

**Table 2:** Altered disease course of all published rNiV_M_ ferret cohorts.

Ferret cohort	Days p.i. to death	Fatality rate	Respiratory signs	Neurological signs	Reference
rNiV_M_-wt	6-8	100%	+++	+	^‡,§^
rNiV_M_-V^ko^	*	0%	−	−	^20^
rNiV_M_-W^ko^	8-11	100%	++	++	^20^
rNiV_M_-C^ko^	8	100%	++	++	^21^
rNiV_M_-C^ko^W^ko^	10-15^†^	60%	+	++/+++□	^21^
rNiV_M_-P_Y116E_	8-9	100%	++	++	^‡^
rNiV_M_-P_Δ116-135_	11-12	100%	+	+++	^‡^

The absence (−) or presence of minor (+), moderate (++), or severe (+++) clinical signs of disease.

^*^All of these animals survived.

^†^Some of these animals survived, this indicates time to death for those that succumbed.

^‡^Data from the current study.

^§^Similar results to other rNiV_M_-wt infected ferrets in refs^20,21^.

One of five animals had minor, temporary neurological signs.

^**□**^The two animals that survived only demonstrated moderate neurological signs while those that succumbed demonstrated severe neurological signs.

## Supplementary information

Supplementary Data
