# Supplementary material for: Antagonism of STAT1 by Nipah virus P gene products modulates disease course but not lethal outcome in the ferret model
Source: Sci Rep. 2019 Nov 13;9:16710. doi: 10.1038/s41598-019-53037-0 (PMC6853903; doi:10.1038/s41598-019-53037-0)
Supplement: Supplementary file 1 — Supplementary Data [file 41598_2019_53037_MOESM1_ESM.pdf]

TITLE

Antagonism of STAT1 by Nipah virus P gene products modulates disease course but not lethal outcome in the ferret model

AUTHORS AND AFFILIATIONS

Benjamin A. Satterfield[1,2,3], Viktoriya Borisevich[1,2], Stephanie L. Foster[1,2], Sergio E. Rodriguez[1,2], Robert W. Cross[1,2], Karla A. Fenton[1,2], Krystle N. Agans[1,2], Christopher F. Basler[4] , Thomas W. Geisbert[1,2], and Chad E. Mire[1,2*]

[1] Galveston National Laboratory, University of Texas Medical Branch, Galveston, TX, USA

[2] Department of Microbiology and Immunology, University of Texas Medical Branch, Galveston, TX, USA

[3] Mayo Clinic, Department of Medicine, Rochester, MN, USA

[4] Center for Microbial Pathogenesis, Institute for Biomedical Sciences, Georgia State University, Atlanta, GA, USA

[*]Corresponding Author: chmire@utmb.edu

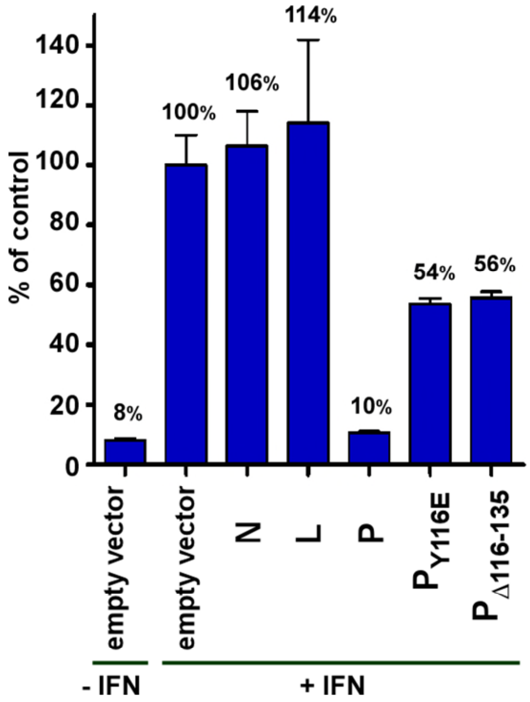

Supplementary Figure 1. Luciferase expression with additional controls. Relative IFN-induced luciferase expression of 293T cells transfected with vector only or various NiV_M expression plasmids after pre-treatment with universal IFN-α or non-treated control as indicated. This is an adjunct to Figure 1b demonstrating that the presence of other NiV_M proteins does not inhibit ISG signaling.

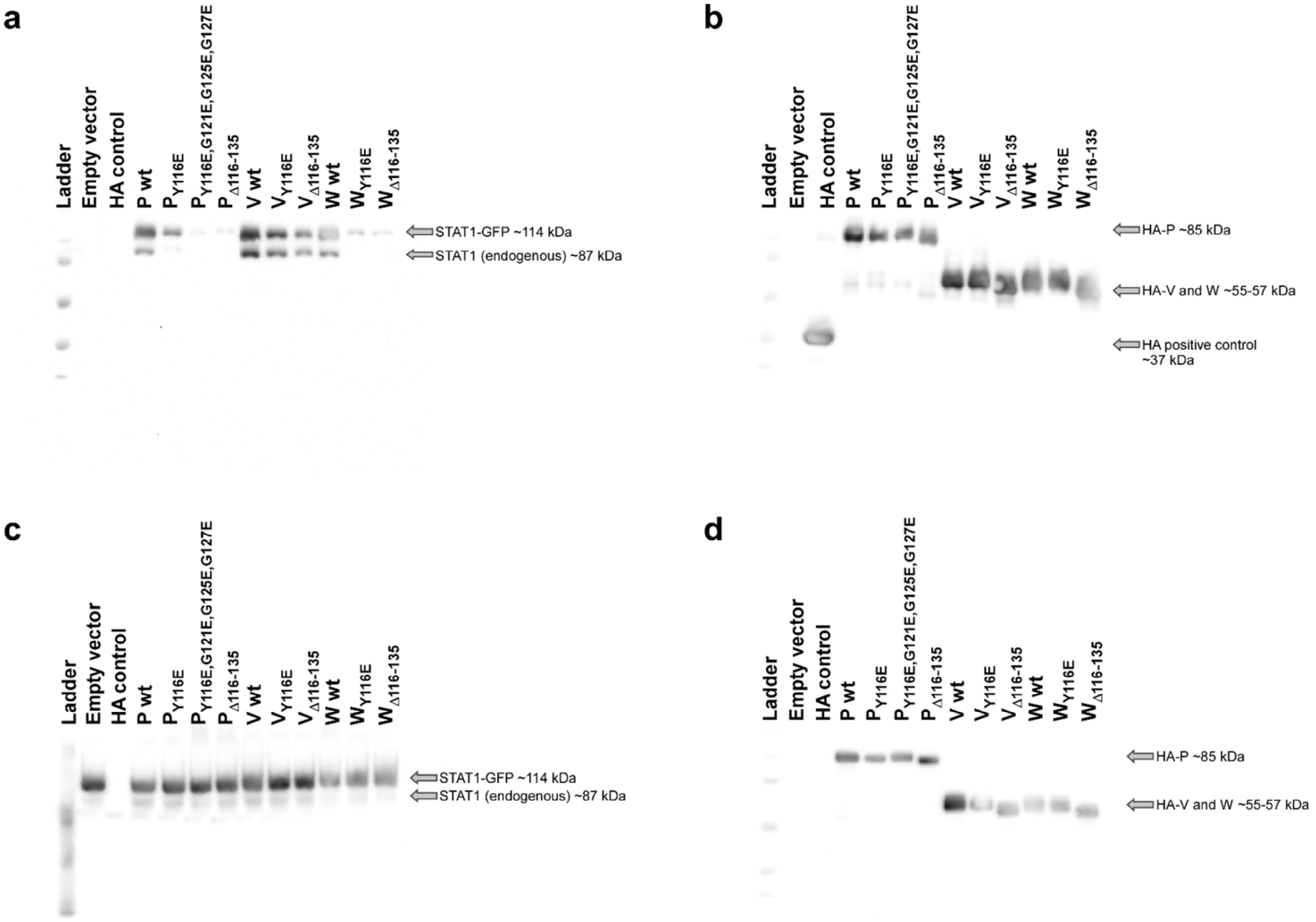

Supplementary Figure 2. Full Western blots characterizing STAT1-binding mutations. As detailed in Figure 2, 293T cells were transfected with pCAGGS-STAT1-GFP along with pCAGGS-HA NiV-P, -V, and -W wild-type and mutant proteins were then assessed at 24 hours post-transfection by Western blot, representative blots from three independent experiments are shown. Relative binding of P, V, and W mutants with STAT1 was assessed using co-immunoprecipitation with HA and stained with **(a)** α-STAT1 or **(b)** α-HA. Similarly, the total amount of expressed protein for P, V, and W mutants is observed in whole cell lysates stained with **(c)** α-STAT1 or **(d)** α-HA.

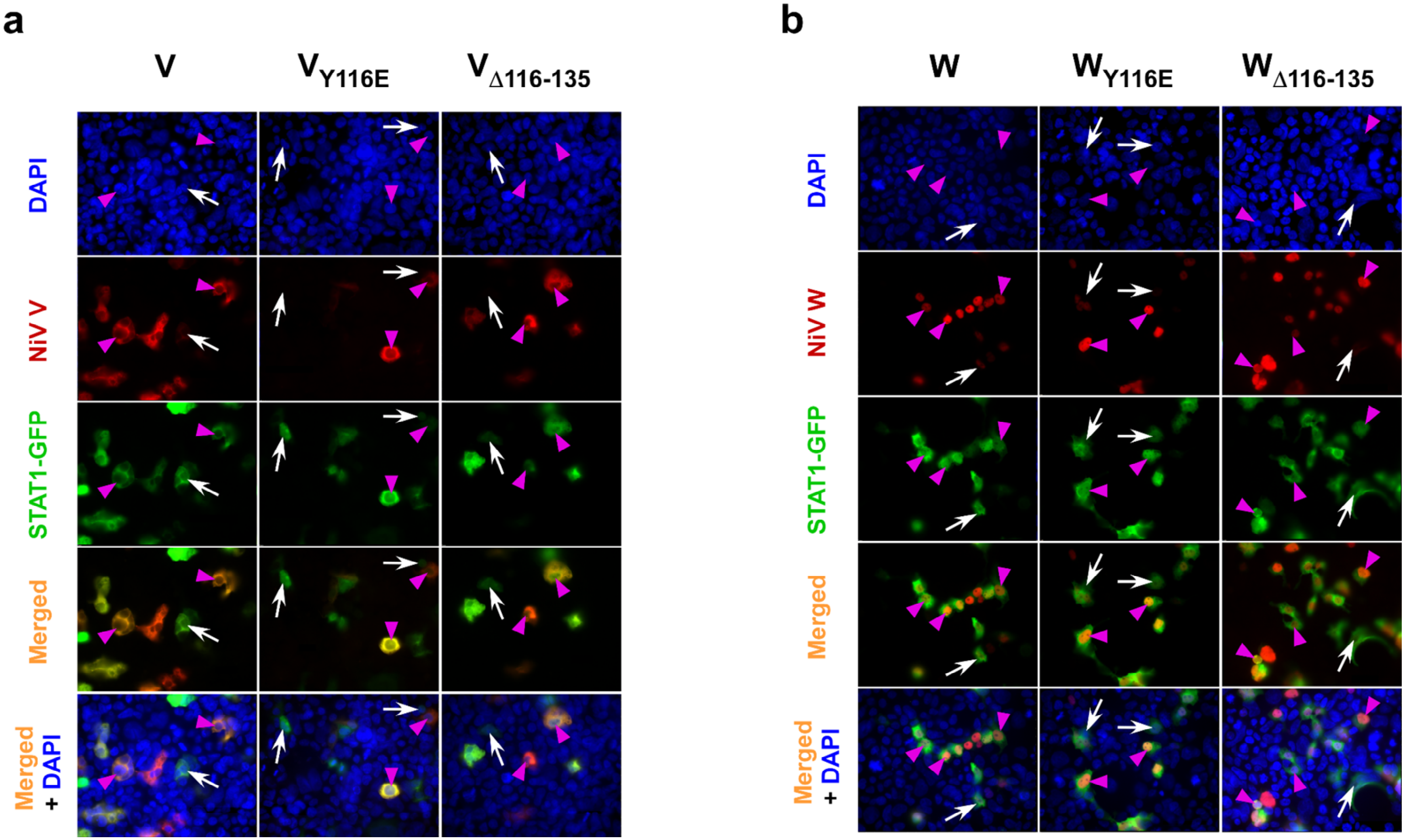

Supplementary Figure 3. Immunofluorescence assay of NiV V, W, and STAT1 proteins in transfected 293T cells. (a) Transfected cells were treated with universal IFN-α then fixed. Cell nuclei are stained with DAPI (blue); cells transfected with HA-tagged NiV V protein (red) show V protein in the cytoplasm but not the nucleus; cells transfected with fused STAT1-GFP show STAT1 in both the cytoplasm and the nucleus when V protein is not co-transfected (white arrows). Cells transfected with STAT1-GFP show no STAT1 present in the nuclei when co-transfected with wild-type NiV V (magenta arrowhead; first column panels) but STAT1 is present when co-transfected with NiV V protein containing the V_{Y116E} mutation (magenta arrowhead; second column panels) or $V_{\Delta 116\text{-}135}$ deletion (magenta arrowhead; third column panels) indicating the ability of these mutations to ablate the ability of NiV V to sequester STAT1. **(b)** Transfected cells were treated with universal IFN-α then fixed. Cell nuclei are stained with DAPI (blue); cells transfected with HA-tagged NiV W protein (red) show W protein in the nucleus; cells transfected with fused STAT1-GFP show STAT1 in both the cytoplasm and the nucleus when W protein is not co-transfected (white arrows). Cells transfected with STAT1-GFP show STAT1 present in the nuclei when co-transfected with wild-type NiV W (magenta arrowhead; first column panels) and W protein containing the W_{Y116E} mutation (magenta arrowhead; second column panels) or $W_{\Delta 116\text{-}135}$ deletion (magenta arrowhead; third column panels) demonstrating the known ability of W protein to sequester STAT1 in the nucleus. Images taken: 20x.

a

NiV$_M$ Genome Nucleotide Position	P ORF Amino Acid Position	C ORF Amino Acid Position
T2751A and C2753G	Y116E	Y108Stop and T109S
G2767A	G121E	E114K
G2779A	G125E	D118R
G2785A	G127E	D120R
T2793G and A2795C	S130A	L122L and Q123P
A2796G and G2797C	S131A	Q123Q and A124P
G2809A and G2810A	G135E	G128R
Δ2766-2795	Δ121-130	Δ114-123
Δ2751-2810	Δ116-135	Δ109-128

b

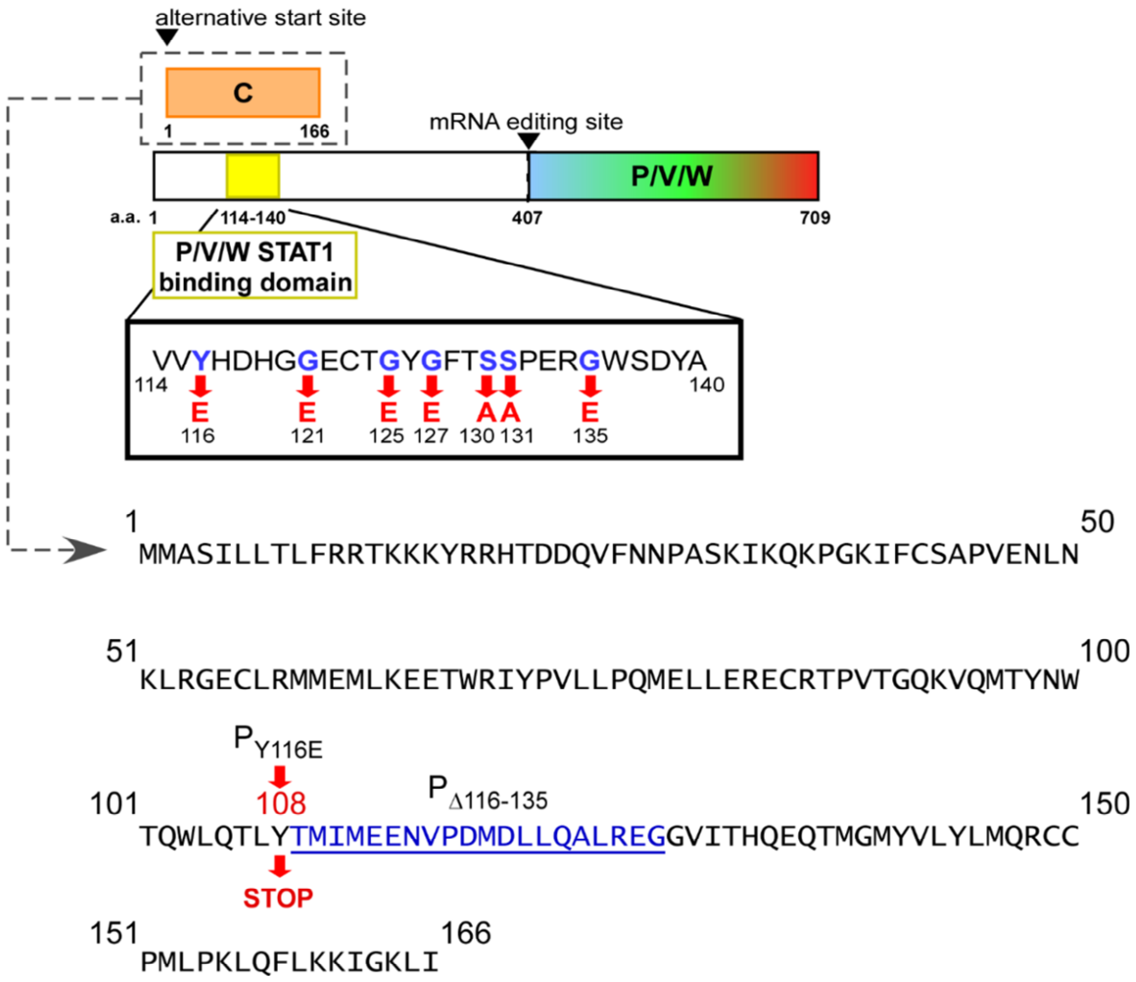

Supplementary Figure 4. STAT1^{blind} mutations utilized in this study. (a) The nucleotide positions and the subsequent amino acid mutations in the P and C ORFs are shown. The original nucleotide/amino acid is shown in blue, the mutated nucleotide/amino acid is shown in green. STOP codon is shown in red. Silent mutations remain in blue. **(b)** The overlap of the P and C ORFs are shown in the upper panel, the amino acid sequence of the C protein is shown below. The position of the P_{Y116E} and $P_{\Delta 116\text{-}135}$ mutations are shown.

```
Range 1: 1 to 750 Graphics                                                      Next Match   Previous Match

Score             Expect  Method                        Identities      Positives       Gaps
1506 bits(3899)   0.0     Compositional matrix adjust.  727/750(97%)    738/750(98%)    0/750(0%)

Query  1    MSQWYELQQLDSKFLEQVHQLYDDSFPMEIRQYLAQWLEKQDWEHAANDVSFATIRFHDL  60
            MSQWYELQQLDSKFLEQVHQLYDDSFPMEIRQYLAQWLEKQDWEHAANDVSFATIRFHDL
Sbjct  1    MSQWYELQQLDSKFLEQVHQLYDDSFPMEIRQYLAQWLEKQDWEHAANDVSFATIRFHDL  60

Query  61   LSQLDDQYSRFSLENNFLLQHNIRKSKRNLQDNFQEDPIQMSMIIYSCLKEERKILENAQ  120
            LSQLDDQYSRFSLENNFLLQHNIRKSKRNLQDNFQEDP+QMSMII +CLKEER+ILENAQ
Sbjct  61   LSQLDDQYSRFSLENNFLLQHNIRKSKRNLQDNFQEDPVQMSMIICNCLKEERRILENAQ  120

Query  121  RFNQAQSGNIQSTVMLDKQKELDSKVRNVKDKVMCIEHEIKSLEDLQDEYDFKCKTLQNR  180
            RFNQAQSG+IQSTVMLDKQKELDSKVRNVKDKVMCIEHEIK+LEDLQDEYDFKCKTLQNR
Sbjct  121  RFNQAQSGSIQSTVMLDKQKELDSKVRNVKDKVMCIEHEIKTLEDLQDEYDFKCKTLQNR  180

Query  181  EHETNGVAKSDQKQEQLLLKKMYLMLDNKRKEVVHKIIELLNVTELTQNALINDELVEWK  240
            EHETNGVAK+DQKQEQLL++KMYLMLDNKRKEVV KIIELLNVTELTQ ALINDELVEWK
Sbjct  181  EHETNGVAKNDQKQEQLLIQKMYLMLDNKRKEVVLKIIELLNVTELTQKALINDELVEWK  240

Query  241  RRQQSACIGGPPNACLDQLQNWFTIVAESLQQVRQQLKKLEELEQKYTYEHDPITKNKQV  300
            +RQQSACIGGPPNACLDQLQNWFTIVAESLQQVRQQLKKLEELEQKYTYEHDPITKNKQ
Sbjct  241  QRQQSACIGGPPNACLDQLQNWFTIVAESLQQVRQQLKKLEELEQKYTYEHDPITKNKQG  300

Query  301  LWDRTFSLFQQLIQSSFVVERQPCMPTHPQRPLVLKTGVQFTVKLRLLVKLQELNYNLKV  360
            LWDRTFSLFQQLIQSSFVVERQPCMPTHPQRPLVLKTGVQFTVKLRLLVKLQELNYNLKV
Sbjct  301  LWDRTFSLFQQLIQSSFVVERQPCMPTHPQRPLVLKTGVQFTVKLRLLVKLQELNYNLKV  360

Query  361  KVLFDKDVNERNTVKGFRKFNILGTHTKVMNMEESTNGSLAAEFRHLQLKEQKNAGTRTN  420
            KVLFDKDVNERNTVKGFRKFNILGTHTKVMNMEESTNGSLAAEFRHLQLKEQKNAGTRTN
Sbjct  361  KVLFDKDVNERNTVKGFRKFNILGTHTKVMNMEESTNGSLAAEFRHLQLKEQKNAGTRTN  420

Query  421  EGPLIVTEELHSLSFETQLCQPGLVIDLETTSLPVVVISNVSQLPSGWASILWYNMLVAE  480
            EGPLIVTEELHSLSFETQLCQPGLVIDLETTSLPVVVISNVSQLPSGWASILWYNMLV E
Sbjct  421  EGPLIVTEELHSLSFETQLCQPGLVIDLETTSLPVVVISNVSQLPSGWASILWYNMLVTE  480

Query  481  PRNLSFFLTPPCARWAQLSEVLSWQFSSVTKRGLNVDQLNMLGEKLLGPNASPDGLIPWT  540
            PRNLSFFL PPCARW+QLSEVLSWQFSSVTKRGLNVDQLNMLGEKLLGPNA PDGLIPWT
Sbjct  481  PRNLSFFLNPPCARWSQLSEVLSWQFSSVTKRGLNVDQLNMLGEKLLGPNAGPDGLIPWT  540

Sbjct  541  RFCKENINDKNFPFWLWIESILELIKKHLLSLWNDGCIVGFISKERERALLKDQQPGTFL  600

Query  601  LRFSESSREGAITFTWVERSQNGGEPDFHAVEPYTKKELSAVTFPDIIRNYKVMAAENIP  660
            LRFSES REGAITFTWVER QNGGEP FHAVEPYTKKELSAVTFPDIIRNYKVMAAENIP
Sbjct  601  LRFSESCREGAITFTWVERPQNGGEPHFHAVEPYTKKELSAVTFPDIIRNYKVMAAENIP  660

Query  661  ENPLKYLYPNIDKDHAFGKYYSRPKEAPEPMELDGPKGTGYIKTELISVSEVHPSRLQTT  720
            ENPLKYLYPNIDKDHAFGKYYSRPKEAPEPMELDGPKGTGYIKTELISVSEVHPSRLQTT
Sbjct  661  ENPLKYLYPNIDKDHAFGKYYSRPKEAPEPMELDGPKGTGYIKTELISVSEVHPSRLQTT  720

Query  721  DNLLPMSPEEFDEVSRIVGSVEFDSMMNTV  750
            DNLLPMSPEEFDEVSRIVGSVEFDSMMN V
Sbjct  721  DNLLPMSPEEFDEVSRIVGSVEFDSMMNAV  750
```

Supplementary Figure 5. Amino acid identity of STAT1 from humans and ferrets. Amino acid sequences of the STAT1 gene from humans (assession number NP_009330.1), listed as “Query,” and ferrets (assession number XP_012917318.1), listed as “Sbjct,” were compared using NCBI’s BLASTp software and found to have 97% amino acid identity.

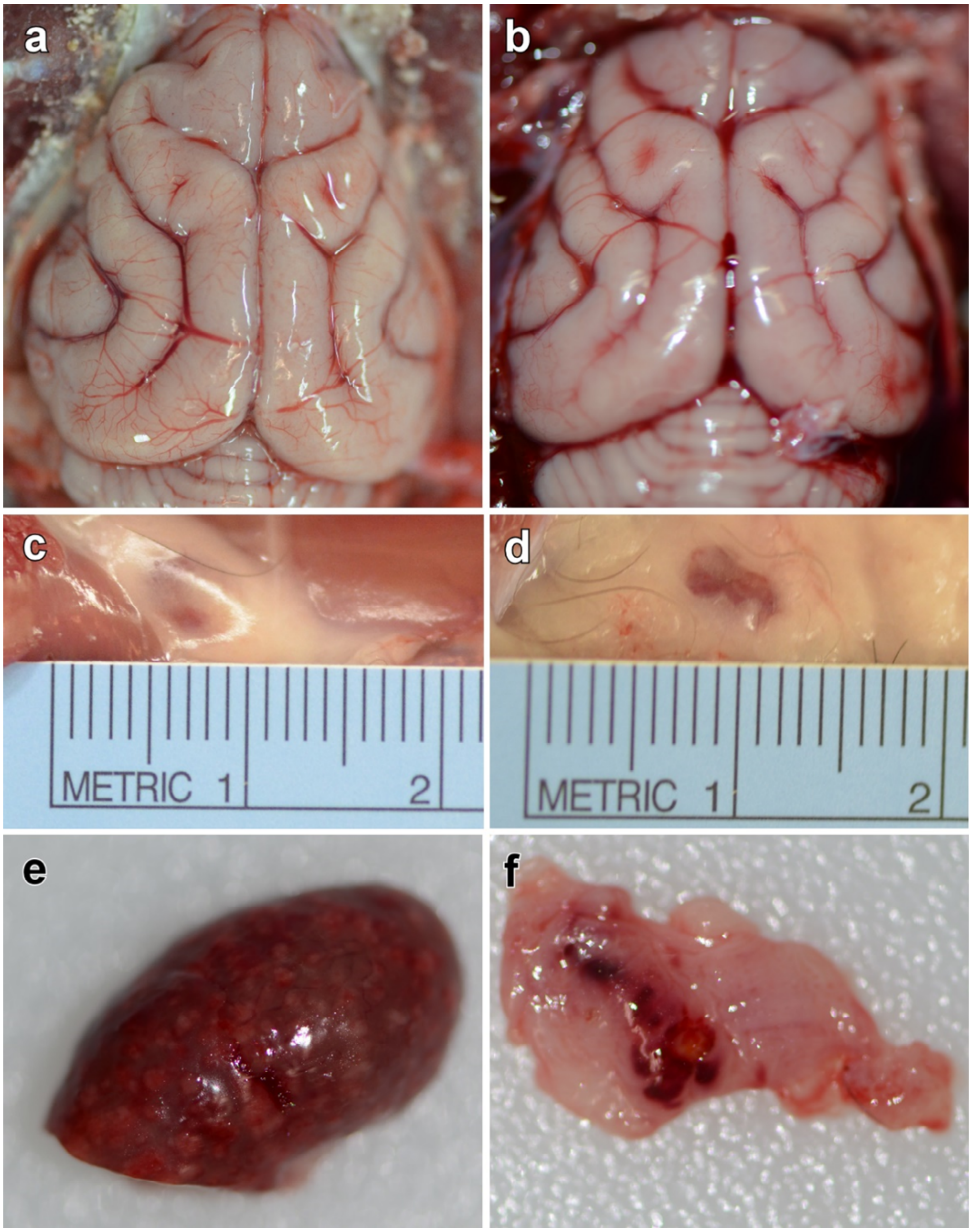

Supplementary Figure 6. Gross pathology of brain, lymph nodes, kidney, and bladder. Representative brains from ferrets infected with $rNiV_M$-P_{Y116E} **(a)** or $rNiV_M$-$P_{\Delta 116\text{-}135}$ **(b)** both show severe congestion of the blood vessels surrounding the brain. Enlarged lymph nodes from the axial **(c)** and inguinal **(d)** regions of a ferret infected with $rNiV_M$-P_{Y116E}. Necrosis and hemorrhage of the kidney was seen in most ferrets from all three cohorts **(e)**. Mucosal hemorrhagic lesions of the urinary bladder were seen in some ferrets from all three cohorts **(f)**.

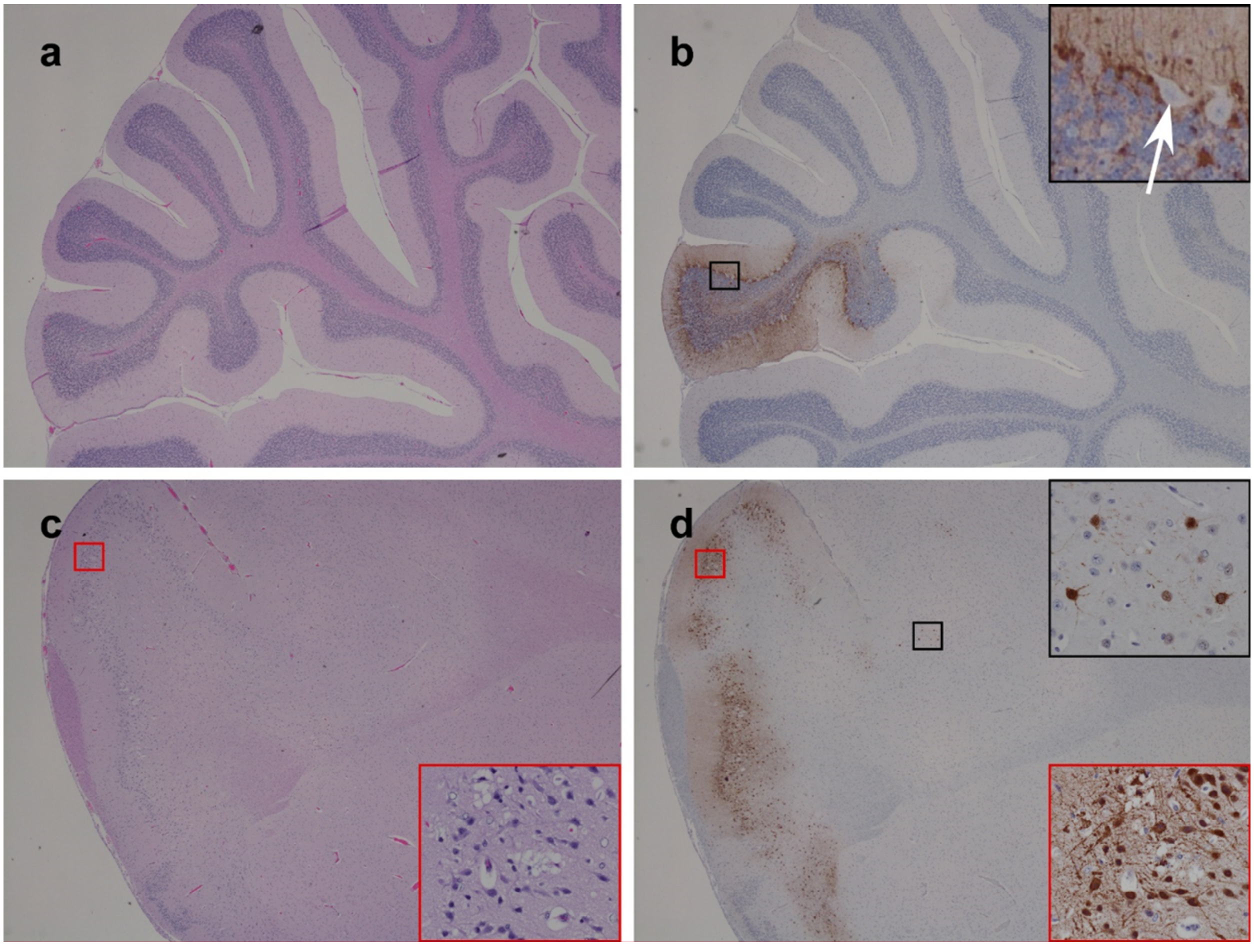

Supplementary Figure 7. H&E and immunohistochemistry of ferret brains. H&E **(a, c)** and immunohistochemistry labeled with a NiV N protein-specific polyclonal rabbit antibody **(b, d)** of the cerebellum of ferret rNiV$_M$-P$_{Y116E}$-05 **(a, b)** and frontal cortex of ferret rNiV$_M$-P$_{Y116E}$-02 **(c, d)**. Insert in panel **b** shows infected neurons with Purkinje cell sparing. Red inserts in panels **c and d** show marked locally extensive, vacuolar plaque with necrosis and gliosis within the grey matter in regions with diffuse, strongly immunopositive neuronal involvement. No vacuolar plaque is observed without associated NiV antigen. Black insert in panel **d** shows scattered deep neuron infection. Images taken: brain 2x.

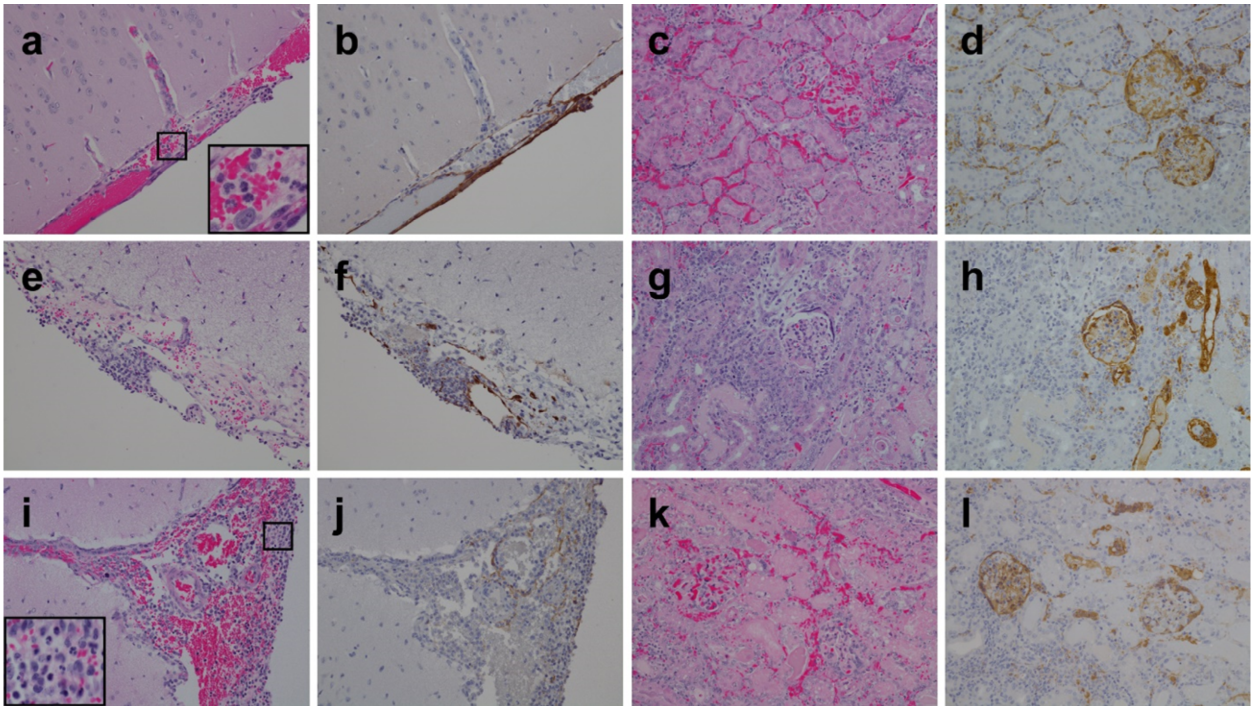

Supplementary Figure 8. H&E and immunohistochemistry of ferret brain meninges. Representative H&E **(a,e,i)** and immunohistochemistry labeled with a NiV N protein-specific polyclonal rabbit antibody **(b,f,j)** of the meninges around the brain from representative ferrets infected with rNiV$_M$-wt **(a,b)**, rNiV$_M$-P$_{Y116E}$ **(e,f)**, and rNiV$_M$-P$_{\Delta 116\text{-}135}$ **(i,j)**. Inserts show mononuclear cell and polynuclear cell infiltration. Representative H&E **(c,g,k)** and immunohistochemistry labeled with a NiV N protein-specific polyclonal rabbit antibody **(d,h,l)** of renal glomeruli of the kidneys from representative ferrets infected with rNiV$_M$-wt **(c,d)**, rNiV$_M$-P$_{Y116E}$ **(g,h)**, and rNiV$_M$-P$_{\Delta 116\text{-}135}$ **(k,l)**. Images taken: brain 20x, kidney 20x.

Supplementary Table 1. Pathology score comparison of selected organs for all published $rNiV_M$ cohorts*														
**Ferret cohort**	**Reference**	**Liver**			**Spleen**			**Lung**				**Brain**		
		**Gross[a]**	**H&E[b]**	**IHC**	**Gross[c]**	**H&E[d]**	**IHC**	**Gross[e]**	**H&E[f]**	**IHC**	**Nodules**	**Gross[g]**	**H&E[h]**	**IHC**
**$rNiV_M$-wt**	§	3	3	3	4	4	3	3	2	2	2	1	1	2
**$rNiV_M$-V^{ko}**	20	0	0	0	0	0	0	0	0	0	0	0	0	0
**$rNiV_M$-W^{ko}**	20	1.5	2.5	2.5	4	3	3	1.5	3	3	3	1	1	2
**$rNiV_M$-C^{ko}**	21	1.5	3	3	3	3†	3	2	1.5	2‡	1.5	1	0	1
**$rNiV_M$-$C^{ko}W^{ko}$**	21	1	1	0	3	2†	1	1	1	0.5‡	1	1	1	1
**$rNiV_M$-P_{Y116E}**	§	3	3	3	4	3.5	3	2	2	2	2	1.5	2□	2□
**$rNiV_M$-$P_{\Delta 116\text{-}135}$**	§	1.5	2	2	3	2.5†	1.5	2	2.5	1.5‡	2.5	1.5	1.5	2.5

Pathology scoring: scale of 0 – 5; 0 = no lesion; 5 = severe lesions; scores are averaged from all animals in each cohort

***** All specimens were reviewed and scored by the same board certified veterinary pathologist for consistency

[a] Reticulation and multifocal pale areas of necrosis

[b] Hepatocellular necrosis, vacuolar change, congestion, sinusoidal leukocytosis, and periportal lymphoplasmacytic infiltrates

[c] Splenomegaly, mottled surface indicating multifocal necrosis

[d] Splenic architectural destruction, germinal center obliteration, and necrosis

[e] Multifocal pinpoint and/or coalescing hemorrhage

[f] Interstitial pneumonia, necrosis of the alveolar septae, syncytial cell formation, edema, and hemorrhage

[g] Congestion of meningeal blood vessels and blood pooling/clots

[h] Meningitis

† Indicates hypercellularity of the red pulp

‡ Indicates immunolabeling predominately associated with inflammatory nodules

□ Indicates that there was great variability among animals, some scoring much higher, others much lower

§ Current study
